# General and Specific Facets of Anxiety: Psychometric Analysis and Impact on Cognitive Performance

**DOI:** 10.3390/bs16050806

**Published:** 2026-05-18

**Authors:** Evgeniia Alenina, Kristina Terenteva, Vladimir Kosonogov

**Affiliations:** 1 Affective Psychophysiology Laboratory, Institute of Health Psychology, HSE University, 190068 Saint Petersburg, Russia; 2 Faculty of Biology and Biotechnology, HSE University, 117418 Moscow, Russia

**Keywords:** anxiety, cognitive performance, reaction time, factor analysis, Stroop task

## Abstract

Anxiety is a multidimensional construct that influences cognitive performance in complex ways, yet its factor structure and domain-specific effects remain unclear. This study examined (1) the psychometric structure of general and specific anxiety measures, (2) their associations with cognitive performance across different domains, and (3) the predictive power of machine learning models in classifying cognitive performance based on specific anxiety in different domains. A two-stage design was employed: Stage 1 (*N* = 500) assessed self-reported anxiety (trait, state, generalized, social, spatial, and math anxiety) via questionnaires, while Stage 2 (*N* = 104) involved a set of experiments measuring cognitive performance (accuracy and reaction time) across numerical, social, spatial, and control tasks. Factor analyses revealed a correlated yet distinct structure. The model treating anxiety measures as independent factors showed the best fit among tested alternatives; however, all CFA models exhibited suboptimal absolute fit indices (TLI/CFI < 0.73). Regression analyses also demonstrated domain-specific effects: after controlling for state and generalized anxiety, trait anxiety showed small but statistically significant positive associations with performance on the social task (OR = 1.03) and spatial task (OR = 1.07). Machine learning models (Random Forest, Decision Trees, SVM) demonstrated limited predictive accuracy, with ensemble methods outperforming linear models. Prediction of reaction time in cognitive tasks, based on anxiety measures, was less powerful, suggesting that non-anxiety factors play a larger role in cognitive performance. These findings highlight the importance of distinguishing between general and domain-specific anxieties in cognitive research and demonstrate the potential of a machine learning approach in modeling anxiety–performance relationships.

## 1. Introduction

Anxiety is a multifaceted construct with diverse manifestations across cognitive domains (Kryza-Lacombe et al., 2024). While extensive research has established the effects of anxiety on cognitive performance (e.g., numerical problem-solving (Dowker et al., 2016) and spatial thinking (Geer et al., 2024)), the precise nature of these relationships remains complex and context-dependent. Prior studies have usually treated anxiety as a unidimensional construct (negative significant association between anxiety and academic achievement, e.g., meta-analysis (Brumariu et al., 2023)). Other studies focused on domain-specific anxieties (e.g., math anxiety and math achievement, (Barroso et al., 2021) or social anxiety and peer friendship quality, (Chiu et al., 2021)) in isolation. Recent work underscores that anxiety is not a unitary construct but interacts dynamically with cognitive control processes (Liu et al., 2024). However, emerging evidence suggests that different anxieties may interact in different ways, influencing cognitive performance with varying degrees of specificity.

For example, some research demonstrated that math anxiety negatively influenced cognitive performance, particularly in numerical contexts (significant effect for reaction time and accuracy in math anxiety condition (Mammarella et al., 2023)). Math anxiety is a prevalent issue characterized by negative emotional responses to numerical tasks (Barroso et al., 2021) and also linked to deficits in numerical processing (Wang et al., 2015). It is associated with various cognitive processes, including attentional control, working memory, and numerical magnitude processing. Specifically, high math anxiety was associated with reduced attentional control, leading to increased cognitive effort and slower task-switching in arithmetic tasks (Szczygieł & Sarı, 2024). The same effect was reported for high math anxiety and mental calculation, with high math-anxious individuals showing reduced cortical activation during numeric processing (González-Gómez et al., 2023). Math-related stimuli (e.g., pictures of math-related operations) may be perceived as affectively salient by individuals with math anxiety; a study by Linares et al. (2025) demonstrated that individuals with math anxiety perceive math-related stimuli as aversive cues, similar to how they process unpleasant low-arousing images (Linares et al., 2025). The relationship between math anxiety and cognitive performance is complex, often involving both direct and indirect effects. At the same time, math anxiety does not significantly moderate the relationship between problem difficulty and cognitive effort, indicating that anxiety may not increase cognitive load in all contexts (Van Den Bussche et al., 2020). Some studies demonstrate that number skills (e.g., counting, number recognition, symbolic number comparison) and spatial skills (spatial processing) are mediated by math test performance (calculation and procedural arithmetic) (Douglas & LeFevre, 2018).

Similarly to math anxiety, spatial anxiety significantly impacts cognitive performance. Spatial anxiety is the fear and apprehension experienced during tasks that require spatial thinking, such as navigation, mental rotation, and visualization (Alvarez-Vargas et al., 2020). This anxiety type can negatively impact daily activities, including driving and navigation, and is linked to broader cognitive and psychological factors (Nori et al., 2023). Spatial anxiety negatively affects performance in spatial tasks, such as mental rotation and navigation, by consuming cognitive resources needed for these tasks (Lauer et al., 2018). High spatial anxiety is associated with lower performance in spatial perception, mental rotation, and spatial visualization tasks compared with low spatial anxiety groups (Vytal et al., 2013). However, the direct relation between cognitive performance and spatial anxiety is not quite clear. E.g., math anxiety has been shown to negatively impact spatial abilities (mental rotation and navigation), which are essential for numerical problem solving (Ferguson et al., 2015; Gibeau et al., 2023). At the same time spatial anxiety negatively interacts with basic number skills (Likhanov, 2017). Spatial anxiety and math anxiety are positively correlated, suggesting a shared underlying mechanism (Pizzie et al., 2025). However, the relationship between spatial anxiety and cognitive performance is not mediated by math anxiety, indicating that these anxieties may operate independently in affecting specific (math and spatial) abilities (Lourenco & Liu, 2023).

Yet another example of a specific anxiety could be social anxiety, which refers to the feeling of discomfort in social interactions (e.g., meeting and talking to people; (Mattick & Clarke, 1998)). In addition, recent research highlights maladaptive usage of mobile phones and avoiding face-to-face communication in socially anxious individuals (Zsido et al., 2021). Research indicates that individuals with high social anxiety tend to perform less accurately in tasks with identifying emotions in faces, which suggests a specific impairment in social signal processing (Dickter et al., 2018). In addition, social anxiety is negatively associated with self-evaluations and performance expectations among primary and secondary school students (Tuschen-Caffier et al., 2011). While social anxiety mostly negatively correlates with cognitive performance (Baez et al., 2023), the relationship is not straightforward. For instance, no differences in social working memory were found for participants with low social anxiety (Tabak et al., 2016). In this vein, emotion recognition accuracy in socially anxious participants did not significantly differ from low social anxiety groups; however, high social anxiety groups reported less confidence in their answers (Folz et al., 2022). At the same time, other studies report decreased accuracy in emotion recognition in social anxiety groups (Uribe & Meuret, 2024). Understanding the factor structure of anxiety measures and their differential associations with cognitive performance is crucial for refining theoretical models and improving predictive accuracy in both clinical and non-clinical settings.

However, it is not quite clear whether math, spatial and social anxieties are associated only with specific tasks or unite into general anxiety, and if they are overlapping or independent concepts. Exploratory and confirmatory factor analyses (EFA/CFA) can clarify whether anxiety is best conceptualized as a general factor, distinct domain-specific factors, or a hybrid structure. For example, an EFA by Cheng et al. (2022) defined math anxiety as an independent factor suggesting that it should be considered a distinct anxiety disorder specific to numerical learning (Cheng et al., 2022). Another study by Malanchini et al. (2017) figured out that spatial, math, and general anxiety are moderately heritable, with genetic factors accounting for 30% to 41% of the variance, indicating that while there are common genetic and environmental influences, each anxiety type also has unique contributors. The interplay between these anxieties suggests a multifactorial construct, both phenotypically and etiologically, emphasizing the need to study anxiety within specific contexts (Malanchini et al., 2017). At the same time, another study demonstrated that eight general and specific anxiety measures loaded onto one factor, explaining 51% of the variance (Likhanov et al., 2026). However, there is a lack of studies which investigate the factor structure of several specific and general anxieties.

Meanwhile, predictive modeling techniques can assess the extent to which anxiety measures explain variance in task performance across different cognitive domains. Math anxiety negatively impacts both math and spatial performance, with individuals high in math anxiety reporting worse spatial skills and a poorer sense of direction (Ferguson et al., 2015). Spatial ability is closely related to mathematical ability, but math anxiety does not moderate this relationship. Instead, trait anxiety interacts with spatial ability to influence math performance (Likhanov, 2017). Spatial anxiety, particularly navigation and manipulation anxiety, mediates gender differences in math anxiety, with females generally reporting higher levels of both math and spatial anxiety (Delage et al., 2022). In early schooling, math and spatial anxiety are domain-specific, with girls showing higher levels of both compared to boys, which may contribute to the gender gap in STEM (science, technology, engineering and mathematics) fields (Commodari & La Rosa, 2021). Spatialization in working memory, such as organizing sequences spatially, is associated with lower math anxiety and better math performance, suggesting cognitive strategies to mitigate anxiety (Lauer et al., 2018). There is a lack of studies which would investigate both math and social anxiety; however, social support and academic flow have been identified as significant predictors of math anxiety, suggesting that positive social interactions and engagement in learning can mitigate anxiety levels (Kim et al., 2023). However, it has yet to be studied how specific anxieties influence specific cognitive task performance.

The present study addresses these gaps by (1) examining the factor structure of multiple anxiety measures (trait, state, general, and domain-specific anxieties) in order to clarify the load of both general and specific factors of anxiety, (2) investigating their unique and combined associations with cognitive performance across numerical, social, spatial, and control tasks, and (3) evaluating the predictive utility of machine learning models in classifying specific task performance based on specific anxiety type in different domains (math, social and spatial). The results could contribute to the growing literature on anxiety–cognition interactions integrating psychometric and predictive modeling approaches. Our study attempted to advance our understanding of how different anxiety dimensions shape cognitive performance, offering methodological and theoretical insights for future research.

## 2. Methods

### 2.1. Participants

A total of 500 respondents (Stage 1, online) participated in the study (M_age_ = 22.74 years, SD = 5.10, 282 females). Of them, 104 participants (Stage 2, in-lab) from the Moscow region (Russia) were invited to participate in the laboratory experiment (M_age_ = 21.89 years, SD = 4.48, 79 females). Invitation criteria were as follows: the control group (*n* = 41) comprised participants with scores ≤ 1 SD above the sample mean on all anxiety questionnaires; the math-anxiety group (*n* = 12) included individuals with Abbreviated Math Anxiety Scale (Hopko et al., 2003) scores > 1 SD above the mean while all other scales remained < 1 SD (M = 27.33, SD = 3.77); the social anxiety group (*n* = 31) consisted of participants with ASC (Appraisal of Social Concerns; Telch et al., 2004) scores > 1 SD above the mean and all other scales < 1 SD (M = 3.28, SD = 0.61); the spatial anxiety group (*n* = 20) included those with SA (Spatial Anxiety questionnaire; Lawton, 1994) scores >1 SD above the mean and all other scales ≤ 1 SD (M = 30.60, SD = 6.40); and the remaining participants (*n* = 396) exhibited either elevated trait anxiety scores (>1 SD) or mixed profiles with multiple questionnaires exceeding 1 SD simultaneously. The research protocol received ethical approval from the university ethical committee (January 2025; No. 118). Informed consent was obtained from all participants prior to their involvement in the study. Online participants were compensated with approximately $10 (adjusted for purchasing power parity), while in-lab participants received $19.

The required sample size for both stages was determined using G*Power v.3.1.9.7 (Faul et al., 2009). The power analysis indicated that a sample of 73 participants with medium effect size (f2 = 0.15, α = 0.05, power (1 − β) = 0.80, with one predictor) would be enough for linear regression models and 67 participants would be sufficient for logistic regression/classification models (Odds Ratio = 2.5 (medium effect size), Pr (Y = 1|X = 0) = 0.5 (baseline probability for balanced binary outcomes)). For machine learning models (e.g., Random Forest, SVM), the sample size was inflated by 20% (N ≈ 88) to account for higher dimensionality and cross-validation requirements. Target enrollment was increased by 15% (N ≈ 84 for regression, N ≈ 77 for classification) to accommodate potential exclusions.

#### Anxiety Questionnaires

The State-Trait Anxiety Inventory (STAI; Spielberger et al., 1983), comprises the Trait Anxiety and the State Anxiety subscales (each of 20 items). For the trait subscale, participants indicate how they ‘generally feel’. Conversely, the state subscale assesses how respondents feel ‘right now’. We utilized a Russian adaptation, which has demonstrated high internal consistency in a large adolescent sample (α = 0.88 for the Trait Anxiety and 0.89 for the State Anxiety; 6). The Generalized Anxiety Disorder 7-item scale (GAD-7; Spitzer et al., 2006) has been designed to evaluate the presence of symptoms associated with generalized anxiety disorder. Participants are asked to reflect on their experiences over the past two weeks. A Russian adaptation, used by us, exhibited high internal consistency (α = 0.85; Likhanov et al., 2026). Appraisal of Social Concerns (Telch et al., 2004) was designed to assess social phobia and consists of 20 items that depict various social situations. We used a Russian adaptation which demonstrated high internal consistency (α = 0.84; Likhanov et al., 2026). The same validated adaptation was used to evaluate math anxiety by Abbreviated Math Anxiety Questionnaire (Hopko et al., 2003) a nine-item questionnaire which describes situations related to processing numerical tasks (α = 0.82; Likhanov et al., 2026). To evaluate spatial anxiety, a 10-item questionnaire was used (Lawton, 1994). Participants rated their anxiety levels in spatially demanding situations—such as navigation, way-finding, mental rotation, and spatial visualization. The current version was translated and back-translated into Russian according to following the International Test Commission guidelines for test translations (International Test Commission, 2018). This process ensured linguistic equivalence and cultural appropriateness of all items and demonstrated high internal consistency (α = 0.86). Descriptive statistics and internal consistency are available in Supplementary Materials (SM)—see Table S1.

### 2.2. Cognitive Experimental Tasks

The Stroop tasks were employed to assess cognitive skills towards neutral (classical Stroop task), numeric, spatial and social stimuli. The classical Stroop task consisted of a series of color words (red, green, blue) presented in various colors that either matched or mismatched the semantic meaning of the words (Richards et al., 1992). The numerical Stroop task consisted of two numbers appearing on the screen which were different in meaning and size. Participants were asked to choose the number which was bigger in size, but not in meaning (Suárez-Pellicioni et al., 2015). The spatial flanker task was employed to assess participants’ spatial attentional control (Lee & Pitt, 2024). Participants were presented with a series of arrows where a central target one was flanked by distractor stimuli. Participants had to indicate the target arrow’s direction. The social Stroop task was utilized to evaluate cognitive skills related to emotion recognition within a social context. This task involved presenting a series of facial expressions (sad, happy, and angry) accompanied by written words “sad”, “happy”, and “angry” (Luciana et al., 2018), adapted by (Mohammed et al., 2022). Overall, there were 80 stimuli for each task type. Forty of them were congruent, while forty were incongruent. Participants were instructed to respond with keyboard buttons as soon as possible, but correctly.

### 2.3. Procedure

**Figure 1:**
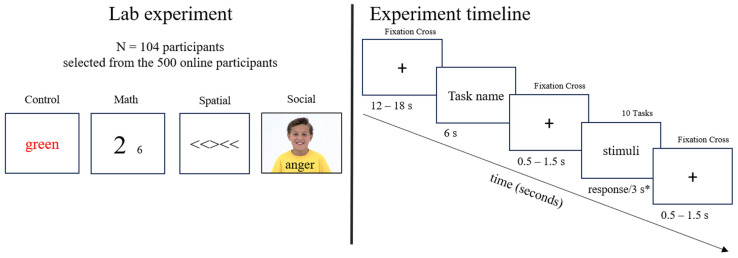
Experiment design. Notes: * response/3 s—participants pressed a key to respond; if the response took more than 3 s, the next stimulus appeared automatically.

All participants were recruited online through the University’s social networks. Interested individuals (*N* = 500) completed an online questionnaire collecting demographic data (age, gender) and assessing anxiety levels (Stage 1). Subsequently, 104 participants were invited to the laboratory experiment (Stage 2), designed and implemented in PsychoPy 2023.2.3, which lasted approximately 1.5–2 h (Figure 1). Participants completed four cognitive tasks: the color Stroop, numerical Stroop, spatial flanker, and social Stroop. Each trial began with a central fixation cross (12–18 s), followed by stimulus presentation (until response or up to 3 s), and an inter-trial interval from 0.5 to 1.5 s. During the practice phase, participants completed 10 trials per task category (color, social, spatial, and numerical) with trial-by-trial correctness feedback (correct/incorrect). Instructions were provided before each block, and only participants who achieved >90% accuracy in practice advanced to the main experimental phase. Those scoring <90% received additional practice rounds (with the same feedback structure) until reaching the ≥90% threshold. In the main phase, participants completed 32 experimental blocks (eight per task category), each containing 10 trials, yielding 80 trials per category and 320 trials in total. Within each block, congruent and incongruent trials were pseudorandomized with a maximum run length of three identical conditions. Prior to each block, a task cue (‘Color’, ‘Numeric’, ‘Spatial’, or ‘Social’) was displayed. Response mapping used the left and right arrow keys and was counterbalanced across participants via a Latin square design. No feedback was provided during the main phase. Blocks were presented in a pseudorandom order, ensuring that no more than three consecutive blocks belonged to the same category. See Figure 1 for more details.

### 2.4. Data Analysis

Data were analyzed using R v. 4.3.1 (libraries ‘lavaan’, ‘psych’, ‘dplyr’, ‘ggcorrplot’, ‘rstatix’) and Python v. 3.11 (packages ‘numpy’, ‘pandas’, ‘sklearn’). Firstly (Stage 1; *N* = 500), correlation analysis of all self-report data was carried out, and the factor structure was explored. Exploratory factor analysis (EFA) was conducted with the oblimin rotation. In confirmatory factor analysis (CFA) five models were explored: 1. A 1-factor ‘General’ model, where all anxiety measures were loaded onto one general factor by items. 2. A 2-factor ‘General and Specific model’, where STAI and GAD-7 were loaded on one factor (as general anxiety), while spatial, math, and social anxiety measures were loaded onto another (as specific anxieties). 3. A 4-factor model, where general, math, social, and spatial anxiety measures were treated as four separate factors. 4. A 1-factor model comprising only STAI and GAD-7 anxiety questionnaires. 5. A 5-factor model, where each questionnaire was treated as a separate factor. CFA assumptions were as follows: RMSEA ranging from 0.06 to 0.08, TLI ≥ 0.95, and CFI ≥ 0.95 (Schreiber et al., 2006).

Secondly (Stage 2; *N* = 104), several models were tested in order to predict specific cognitive performance based on specific anxiety scores, with trait and state anxiety as covariates due to their high correlation (about 0.70). Logistic regression models were applied for cognitive performance, measured as correct responses with a binary scale (correct\incorrect). Then linear regression models were used for reaction time of cognitive performance. To predict cognitive performance from different anxiety scores, four models for both regressions were applied. Model 1: numeric performance was predicted from math anxiety score; Model 2: spatial task was predicted from spatial anxiety; Model 3: face recognition was predicted from social anxiety score;. Model 4: color task was predicted from general anxiety. Although 5680 raw trials were collected during the experimental phase, all dependent variables were aggregated to the participant level prior to statistical modeling (mean reaction times and summed accuracy per task). The analytical dataset therefore comprised *N* = 104 independent observations (one per participant). This aggregation prevents pseudo-replication and within-subject dependence in the regression and machine learning models. To further safeguard against data leakage, all train-test splits and cross-validation procedures were performed at the participant level. Consequently, the data partitioning was applied to participant-level rows, ensuring that no individual’s data appeared in both training and testing subsets. This approach evaluates the model’s ability to generalize to unseen individuals rather than to unseen trials from the same individual, aligning the unit of analysis with the unit of power estimation (*N* = 104 participants).

Machine learning analyses were conducted using the Scikit-Learn Python package (version 0.23.2) to predict cognitive performance from questionnaire data. To prevent data leakage, the analytical dataset was partitioned at the participant level into training (70%) and testing (30%) subsets. Five-fold cross-validation was applied on the training set for hyperparameter optimization and model selection.

For classification models predicting accuracy, class imbalance was addressed using automatically balanced class weights (proportional to inverse class frequencies), as correct responses predominated (mean accuracy = 0.91, range 0.78–0.97 across tasks). The following algorithms and fixed hyperparameter configurations were employed: K-Nearest Neighbors (k = 20, distance-weighted voting); Linear Support Vector Classifier (L2 penalty, Crammer–Singer multiclass strategy, balanced class weights, standard convergence tolerance); Random Forest (1000 estimators, entropy splitting criterion, max_depth = 3, max_features = 0.6, class_weight = ‘balanced_subsample’); and Decision Tree (Gini impurity criterion, max_depth = 5, max_features = 1.0, min_samples_split = 2, min_samples_leaf = 1, balanced class weights). For regression models predicting reaction time, the same algorithmic family was adapted to capture potential nonlinear relationships, given that linear models accounted for <1% of variance (R^2^ ≤ 0.009). Predictors were structured into task-specific, general, and combined anxiety sets. Model performance was evaluated using Mean Absolute Error (MAE) and Root Mean Square Error (RMSE), which provide stable error estimates for continuous outcomes. Negative R^2^ values, observed in some models, indicate performance inferior to a naive mean baseline—a recognized outcome when predictors explain minimal variance in noisy data. Such cases were flagged and excluded from primary interpretation.

## 3. Results

### 3.1. Correlation and Factor Structure of Anxiety Measures

Descriptive statistics for anxiety questionnaires are detailed in Supplementary Materials, Table S1. Correlation analysis revealed that all self-report measures exhibited positive correlations. Specifically, correlation coefficients ranged from small for Spatial and State Anxiety (r = 0.29, *p* < 0.05) to high for Trait and State Anxiety (r = 0.72, *p* < 0.05).

Exploratory factor analysis (EFA) with oblimin rotation was conducted to examine the underlying structure of anxiety items. Sampling adequacy was excellent (KMO = 0.94), and Bartlett’s test of sphericity was significant, χ^2^(2329) = 3261, *p* < 0.001, indicating that the correlation matrix was suitable for factor extraction. Factor retention was guided by the Kaiser criterion (eigenvalues > 1) and scree plot inspection, which together suggested a multi-factor solution. A 16-factor solution was retained, explaining 55% of the total variance. Additional indices supported the adequacy of the solution: RMSR = 0.02, RMSEA = 0.02, and BIC = −11,213.16.

A separate EFA using total scores (sums/means of the questionnaires) yielded a one-factor solution (KMO = 0.84), explaining 52% of the variance. However, the item-level analysis provided greater granularity for examining domain-specific variance and was therefore retained for subsequent analyses. For detailed factor loadings, see Supplementary Materials Table S2a (items) and Table S2b (total scores).

Table 1 represents the results of CFA. Five competing CFA models were tested to examine the factor structure of anxiety measures: (1) General model (1-factor): all items from all six questionnaires (STAI-T, STAI-S, GAD-7, AMAS, ASC, SAQ) loaded onto a single common factor, representing a unitary anxiety construct. (2) General and Specific model (2-factor): items from STAI-T, STAI-S, and GAD-7 loaded onto a ‘General anxiety’ factor, while items from AMAS, ASC, and SAQ loaded onto a ‘Domain-specific anxiety’ factor. (3) Four-factor model: items loaded onto four correlated factors representing General anxiety (STAI-T/S + GAD-7), Math anxiety (AMAS), Social anxiety (ASC), and Spatial anxiety (SAQ). (4) General distress model (STAI-T/S + GAD-7; 1-factor): only items from STAI-T, STAI-S, and GAD-7 loaded onto a single factor, excluding domain-specific measures. (5) All measures independently (6-factor): each of the six subscales (STAI-T, STAI-S, GAD-7, AMAS, ASC, SAQ) was modeled as a separate correlated factor. Model fit was evaluated using RMSEA (acceptable: 0.06–0.08), TLI (≥0.95), and CFI (≥0.95) following Schreiber et al. (2006). Additional indices (AIC, BIC, χ^2^) were used for relative model comparison. Additional factor loadings are presented in Supplementary Materials (Table S2b).

In addition, all confirmatory and exploratory factor analyses employed Maximum Likelihood estimation. Although questionnaire items are ordinal, simulation studies indicate that standard ML performs adequately and yields stable factor loadings and fit indices when scales have ≥4 response categories and sample sizes are moderate to large (Rhemtulla et al., 2012). Given our 4–5 point Likert scales and *N* = 500, ML estimation was appropriate.

### 3.2. Regression Models

To investigate relationships between anxiety and cognitive performance, we tested a series of regression models (Table 2). The correlations between anxiety measures and cognitive outcomes, specifically correct responses and reaction times, are illustrated in Supplementary Materials Figure S2a,b.

The results revealed different patterns between anxiety measures and cognitive performance (accuracy) across domains. For numerical task performance, neither math anxiety (AMAS) nor state/trait anxiety (STAI) showed significant effects (OR = 1.00, *p* = 1.00). In contrast, emotion recognition performance demonstrated a small but significant positive association with trait anxiety (OR = 1.03, *p* = 0.002), while specific social and state anxiety showed null effects. Spatial reasoning revealed different effects: trait anxiety also enhanced performance (OR = 1.07, *p* < 0.001), while the specific spatial anxiety impaired performance (OR = 0.96, *p* = 0.009). For the control task we found no significant associations between any general anxiety (GAD, STAI-T, STAI-S) and color processing outcomes (*p* > 0.05).

Linear regressions were then performed to examine the relations between self-reported anxiety and reaction times in cognitive tasks. The same four models (with domain-specific anxiety predictors and trait/state anxieties) were used. The results of the analysis demonstrated domain-specific anxiety effects on cognitive performance (Table 3). Math and spatial anxiety showed small associations with the respective tasks, (anxiety led to a slower solving), while social anxiety had no impact. Trait anxiety exerted the most consistent influence, improving performance in all tasks, while state anxiety improved only spatial tasks. All models reached statistical significance (*p* < 0.001) but explained ≤1% of variance (R^2^ ≤ 0.009), indicating small effects. The opposing patterns for trait anxiety across domains suggest its cognitive consequences are task-dependent. See Table 3 for more details.

### 3.3. Machine Learning Models

The results of prediction quality for correct responses (accuracy) based on self-reported anxiety can be found in Table 4. For numerical tasks, Linear SVC demonstrated superior performance among specific anxiety models, achieving the highest accuracy (60.5%) and F1-score (0.630). Several models demonstrated AUC values below 0.50 as unstable estimates, likely reflecting weak signal-to-noise ratios rather than systematic inversion, and were not used to draw primary conclusions about predictive capability. For social tasks, Model 3 (all anxiety scales) with the KNN algorithm showed the best performance, reaching 62.3% accuracy and an F1-score of 0.655. Random Forest models consistently delivered reliable results across different predictor sets, with AUC values around 0.63, indicating fair classification capability for emotion recognition tasks. For spatial tasks, Decision Tree models emerged as top performers, particularly in the specific anxiety category (Model 1) where they achieved the highest F1-score (0.701) and strong AUC (0.690). Random Forest models also showed strong results, with F1-scores ranging from 0.635 to 0.682 across different predictor combinations, demonstrating consistent effectiveness for spatial ability prediction. For color tasks, Random Forest models excelled, particularly in Model 3 (all scales) which produced the highest AUC (0.746). The Model 1 (specific) Random Forest model achieved the best balance with 62.1% accuracy and an F1-score of 0.621. For more details see Table 4.

Decision Tree and Random Forest consistently outperformed other algorithms across most tasks, particularly for spatial and color tasks, where they achieved the highest metrics. Model 1 (specific anxiety) demonstrated strong performance in spatial tasks (F1-score = 0.701), while Model 3 (all anxieties) excelled in social tasks (F1-score = 0.655). Math tasks were the most challenging to predict (F1-score = from 0.400 to 0.630), whereas spatial tasks yielded the highest overall performance (F1-score = from 0.571 to 0.701). Overall, ensemble methods (Random Forest, Decision Tree) demonstrated the best predictions, while other variations highlighted the importance of model selection based on data characteristics.

The results of prediction quality for reaction time based on anxiety can be found in Table 4. For numerical tasks, SVR demonstrated the strongest performance in Model 3 (all anxieties), achieving the lowest MAE (0.108 s) and highest R^2^ (0.179). For social tasks, prediction errors were relatively high across all models (MAE > 0.149 s), with Random Forest and Decision Tree achieving the best R^2^ values (0.128). SVR achieved the best results for Model 3 (all anxieties) in spatial tasks (MAE = 0.354 s, R^2^ = 0.138). Color tasks showed more consistent results across models, with Random Forest and Decision Tree performing similarly regardless of predictors (MAE ≈ 0.195 s, R^2^ ≈ 0.200). Algorithm performance demonstrated a consistent pattern: SVR achieved the lowest errors when data was properly scaled, particularly for numeric (MAE = 0.108 s) and spatial tasks (MAE = 0.354 s). Random Forest and Decision Tree showed more stable performance across all tasks compared to KNN, which demonstrated particular sensitivity to feature scaling and predictor choice. However, the choice of predictor influenced results in some cases. For example, for numerical tasks, Model 3 (all anxieties) improved R^2^ to 0.179 compared to Model 1 (specific anxieties; R^2^ = 0.005). For other tasks, additional predictors provided minimal benefits. General anxiety predictors sometimes outperformed task-specific predictors. The MAE for models’ performance revealed that prediction errors ranged from 0.11 to 0.49 s depending on tasks, with spatial tasks being particularly challenging to model accurately. For some cases, negative R^2^ values (e.g., spatial tasks in Model 1 with KNN: R^2^ = −0.193) indicated models that performed worse than a simple mean baseline.

Overall, the analysis revealed distinct patterns in model performance across different cognitive tasks. Model 1 (specific anxieties) performed best for spatial and color tasks in terms of accuracy (F1 = 0.701 for spatial and 0.621 for color) and demonstrated the strongest linear predictive capability for reaction time in the color task (R^2^ = 0.200; MAE = 0.196 s). Model 2 (general anxieties) showed an advantage for spatial tasks, with Random Forest achieving F1 = 0.682 for accuracy and stable reaction time predictions (R^2^ = 0.143; MAE = 0.162 s). Model 3 (all anxieties) provided a clear improvement for numerical tasks, where SVR achieved the best R^2^ = 0.179 and lowest MAE = 0.108 s. However, Model 2 (general anxieties) and Model 3 (all anxieties) consistently outperformed Model 1 (specific anxieties); in addition, Model 3 performed slightly better than Model 2 overall. To sum up, no single model demonstrated clear superiority across all domains, suggesting that optimal model selection depends on the specific cognitive task and performance metric.

## 4. Discussion

The present study investigated the complex relationships between different forms of self-reported anxiety and cognitive performance across multiple domains. Our findings reveal several important patterns that could contribute to the existing literature on anxiety–cognition interactions while raising new questions for future research.

The factor analyses yielded several noteworthy findings. The EFA failed to reveal a unifactorial anxiety structure, with items clustering primarily within their respective theoretical domains. This pattern suggests that while different anxiety measures share some common variance, they largely maintain their domain-specific characteristics. The findings are not in line with previous studies. Some studies reported a general factor associated with anxiety sensitivity, which is the fear of anxiety-related sensations in general due to beliefs about their harmful consequences (Murphy et al., 2024). Hence, a general factor, termed ‘negative affectivity’, seems to account for a significant portion of the variance in responses, suggesting that it captures a broad spectrum of emotional distress toward different stimuli (Ghisi et al., 2016). However, even if there is evidence for the dominance of the general anxiety factor, specific factors can provide additional insights into particular concerns, such as cognitive concerns (Do et al., 2022). We acknowledge that extracting 16 factors exceeds the six theoretically expected constructs. This likely reflects item-level specificity, method variance (e.g., shared wording and response formats within questionnaires), and cross-loadings rather than 16 distinct psychological dimensions. Items from the same questionnaire tended to cluster together, suggesting that at the item level, anxiety measures maintain domain-specific characteristics with limited overlap. These results should be interpreted as exploratory.

The confirmatory factor analyses revealed that the 6-factor model—treating each anxiety measure (STAI-T, STAI-S, GAD-7, AMAS, ASC, SAQ) as a distinct, correlated factor—demonstrated a better fit than the tested alternatives (AIC = 105,232.79, BIC = 106,020.92, RMSEA = 0.058). However, we acknowledge that absolute fit indices remained suboptimal (TLI = 0.718, CFI = 0.725), falling below conventional thresholds for adequate model fit (Schreiber et al., 2006). Consequently, these findings should be interpreted as exploratory rather than confirmatory: they suggest that anxiety measures maintain domain-specific characteristics with limited overlap at the item level, but do not provide definitive evidence for a six-factor structure.

This pattern aligns with conceptualizations of anxiety as consisting of both general and specific components (Reise et al., 2013). The general component is often linked to a broad internalizing or distress factor that encompasses a wide range of emotional disorders and accounts for a significant portion of the variance in anxiety symptoms, being associated with general distress and negative emotionality (Gogol et al., 2016). For example, social anxiety and negative/positive affects have been conceptualized as general factors, highlighting their common emotional underpinnings (Kotov, 2006). At the same time, our findings are consistent with previous studies illustrating that specific components of anxiety capture unique symptomatology not explained by the general factor (Drost et al., 2012), supporting the notion of independent elements within the broader anxiety construct (Malanchini et al., 2017). The weak absolute fit of all tested CFA models may reflect several factors: complex cross-loadings between general and specific anxiety items, method variance due to shared wording and response formats within questionnaires, or limitations of traditional CFA in capturing the multidimensional, context-dependent nature of anxiety.

Our results demonstrated that the impact of anxiety on cognition varies substantially in all anxiety types and cognitive domains. The finding that trait anxiety enhanced performance in emotion recognition and spatial reasoning tasks is particularly intriguing. This pattern aligns with the attentional control theory (Eysenck et al., 2007), which posits that anxiety can improve performance on tasks requiring sustained attention. The beneficial effects may stem from increased vigilance and preparatory attention associated with trait anxiety (Bishop, 2009).

Conversely, domain-specific anxieties showed more selective impairment effects. In sum, while numeric and color task performance (accuracy) was not related to self-reported anxiety, trait anxiety showed significant positive effects on face and spatial processing, and spatial anxiety was negatively related to spatial task performance. Spatial anxiety uniquely predicted worse performance on spatial tasks, consistent with previous findings that situation-specific anxieties can disrupt performance in their respective domains (Ashcraft & Moore, 2009). The absence of significant effects for math anxiety on numerical tasks was somewhat surprising, though this may reflect our use of a relatively simple numerical Stroop task rather than more complex everyday and arithmetic problems.

The analyses of cognitive performance in reaction time revealed a more complex pattern. While math anxiety slowed responses in numerical tasks, trait anxiety was associated with faster responses across multiple domains. These opposing effects may reflect different underlying mechanisms: domain-specific anxieties could induce hesitation and repeated checking (Ashcraft & Kirk, 2001), whereas trait anxiety might promote a speed–accuracy tradeoff or avoidance motivation (Derakshan & Eysenck, 2009). The finding that state anxiety specifically facilitated spatial task performance suggests that transient anxiety states may have distinct effects from trait anxiety, possibly by enhancing spatial alertness systems (Cornwell et al., 2011).

The machine learning analyses provided additional nuances to the findings. The superior performance of tree-based algorithms (particularly for spatial and color tasks) suggests that anxiety–performance relationships are likely nonlinear and may involve complex interactions between different anxiety types. However, the generally modest prediction accuracies underscore that anxiety measures alone provide limited information about cognitive performance. This aligns with recent calls for more comprehensive models that would incorporate additional cognitive and affective variables (Pacheco-Unguetti et al., 2010). These nonlinear relations are in line with previous studies. In a longitudinal study by Daker et al. (2021), math anxiety predicted math-related academic achievement independently from math abilities. This suggests that math anxiety effects on performance are complex and likely operate via mechanisms other than negatively affecting math ability (Daker et al., 2021). General anxiety, while correlated with math anxiety, does not directly predict math performance. Its influence is more related to general emotion regulation and response to academic performance rather than to specific cognitive tasks (Field et al., 2019). In addition, our findings support the effects of spatial anxiety on Flanker task performance, specifically associated with increased reaction time congruency effects, impacting cognitive control during the task (Lutz et al., 2025).

### Limitations and Future Directions

Several limitations should be noted. First, our sample consisted primarily of young adults, limiting generalizability to other age groups. Second, the laboratory setting may not fully capture real-world anxiety effects considering selection bias. Third, we focused on self-report measures; future studies could benefit from incorporating physiological indices of anxiety. Fourth, supplementary analyses (see Supplementary Materials) revealed that the cognitive tasks did not differ in congruency effect, as delta scores (incongruent minus congruent) did not significantly differ across anxiety groups. This suggests that the Stroop and Flanker paradigms may have been insufficiently challenging for this sample.

Finally, although we aggregated trial-level data to participant-level metrics and employed participant-level train-test splits to prevent pseudo-replication and data leakage, this approach may obscure subtle within-person dynamics.

Future research should examine anxiety–cognition relationships across more complex cognitive tasks; investigate neural mechanisms underlying these effects; explore potential moderators such as working memory capacity; and develop more sophisticated predictive models incorporating multiple anxiety dimensions. In addition, future research should employ parallel analysis, minimum average partial (MAP) tests, or bifactor/Exploratory Structural Equation Modeling (ESEM) approaches to refine anxiety factor structure and robust estimators (e.g., MLR) or polychoric-based methods (e.g., WLSMV) could be explored in future work with larger samples. Moreover, future research could employ hierarchical modeling (e.g., GLMMs) or trial-level machine learning with strict participant-aware cross-validation to simultaneously capture within- and between-person effects.

While several regression models yielded statistically significant associations between anxiety measures and cognitive performance, the proportion of variance explained was consistently small (R^2^ ≤ 0.009, i.e., <1%). This indicates that although anxiety may show reliable directional associations with task performance in large samples, its practical utility for predicting individual cognitive outcomes is limited. Statistical significance in this context reflects precision of estimation rather than magnitude of effect. Consequently, findings should be interpreted as evidence of weak but systematic relationships, not as support for strong predictive or intervention applications. Future research with larger samples, more sensitive paradigms, or multimodal predictors may be needed to identify anxiety–cognition associations with meaningful practical utility.

## 5. Conclusions

Overall, our findings have several important theoretical implications. First, they support multidimensional models of anxiety that distinguish between general and domain-specific components. Second, they suggest that effects of anxiety on cognition depend critically on both the type of anxiety and the nature of the cognitive task. Third, they highlight the value of examining both accuracy and reaction time measures, as these can reveal distinct patterns of anxiety influence. The study advances our understanding of how different anxiety dimensions relate to cognitive performance. The findings highlight the importance of considering both general and domain-specific anxieties, as well as the particular cognitive demands of different tasks. Trait anxiety shows very small positive associations with certain cognitive tasks, but the overall exploratory power of anxiety for cognitive performance is limited. The results underscore the need for more nuanced theoretical models that could account for these complex interactions.

## Figures and Tables

**Table 1:** CFA fit indices for five tested models of anxiety structure.

Models	AIC	BIC	χ^2^	RMSEA	TLI	CFI
Model 1 General (all in one)	109,986.351	110,711.264	14,396.663	0.078	0.498	0.509
Model 2 General & Specific	107,874.242	108,603.369	12,282.554	0.070	0.596	0.605
Model 3 General & Math & Social & Spatial	106,024.392	106,774.592	10,422.704	0.062	0.681	0.689
Model 4 Trait	67,618.040	68,098.505	7160.890	0.085	0.573	0.588
Model 5 All measures independently	105,232.792	106,020.924	9613.104	0.058	0.718	0.725

Note: General (all in one) = 1-factor model with all items from STAI-T, STAI-S, GAD-7, AMAS, ASC, and SAQ loading onto a single factor. General & Specific = 2-factor model: Factor 1 (General: STAI-T/S + GAD-7), Factor 2 (Specific: AMAS + ASC + SAQ). Four-factor = General (STAI-T/S + GAD-7), Math (AMAS), Social (ASC), and Spatial (SAQ). All measures independently = 6-factor model with each scale (STAI-T, STAI-S, GAD-7, AMAS, ASC, SAQ) as a separate correlated factor. AIC = Akaike Information Criterion; BIC = Bayesian Information Criterion; RMSEA = Root Mean Square Error of Approximation; TLI = Tucker–Lewis Index; CFI = Comparative Fit Index.

**Table 2:** Regression predicting cognitive performance (accuracy) based on self-report anxiety.

Cognitive Performance	Self-Report Scales as Predictors	Odds Ratio [95% CI]	*p*	Model Fit
Numeric Task	Math Anxiety	1.00 [0.96, 1.04]	1.000	0.0
	Trait Anxiety	1.00 [0.98, 1.03]	1.000	
	State Anxiety	1.00 [0.98, 1.02]	1.000	
Social Task	Social Anxiety	0.997 [0.83, 1.20]	0.971	25.3 *
	Trait Anxiety	1.03 [1.01, 1.05]	0.002 **	
	State Anxiety	1.00 [0.98, 1.02]	0.729	
Spatial Task	Spatial Anxiety	0.96 [0.94, 0.99]	0.009 **	47.2 ***
	Trait Anxiety	1.07 [1.04, 1.10]	<0.001 ***	
	State Anxiety	1.01 [0.98, 1.04]	0.530	
Color Task	Generalized Anxiety	1.03 [0.95, 1.12]	0.469	13.1
	Trait Anxiety	1.02 [0.99, 1.05]	0.138	
	State Anxiety	1.01 [0.98, 1.04]	0.472	

Note: *N* = 104 participants. Performance metrics (accuracy sums, mean reaction times) were aggregated from a total of 5680 raw trials prior to analysis. Therefore, all regression and machine learning models were estimated at the participant level. * *p* < 0.05, ** *p* < 0.01, *** *p* < 0.001.

**Table 3:** Linear regression to predict cognitive performance (reaction time) from self-report anxiety.

Model	Predictor	b	β	95% CI	df
Math Task	Math Anxiety	0.003 **	0.045	[0.001, 0.005]	3.15
	Trait Anxiety	−0.003 ***	−0.093	[−0.004, −0.002]	−4.93
	State Anxiety	0.000	−0.011	[−0.001, 0.001]	−0.64
Spatial Task	Spatial Anxiety	0.002 *	0.034	[0, 0.003]	2.35
	Trait Anxiety	−0.002 *	−0.041	[−0.003, 0]	−2.19
	State Anxiety	−0.002 **	−0.048	[−0.003, 0]	−2.66
Social Task	Social Anxiety	−0.007	−0.008	[−0.037, 0.023]	−0.47
	Trait Anxiety	−0.006 ***	−0.073	[−0.009, −0.003]	−3.59
	State Anxiety	0.000	0.002	[−0.002, 0.003]	0.14
Control Task	Generalized Anxiety	−0.005 *	−0.043	[−0.009, −0.001]	−2.41
	Trait Anxiety	−0.003 ***	−0.071	[−0.004, −0.001]	−3.78
	State Anxiety	0.000	0.014	[−0.001, 0.002]	0.73

Note: *N* = 104 participants. Performance metrics (accuracy sums, mean reaction times) were aggregated from a total of 5680 raw trials prior to analysis. Therefore, all regression and machine learning models were estimated at the participant level; Model 1 (math task): R^2^ = 0.009, Adj. R^2^ = 0.008, F(3, 5676) = 16.87, *p* ≤ 0.001; Model 2 (spatial task): R^2^ = 0.006, Adj. R^2^ = 0.005, F(3, 5676) = 10.98, *p* ≤ 0.001; Model 3 (social task): R^2^ = 0.006, Adj. R^2^ = 0.005, F(3, 5676) = 10.61, *p* ≤ 0.001; Model 4 (control task): R^2^ = 0.009, Adj. R^2^ = 0.008, F(3, 5676) = 16.63, *p* ≤ 0.001. Higher reaction time means worse performance. * *p* < 0.05, ** *p* < 0.01, *** *p* < 0.001.

**Table 4:** Prediction quality for correct responses (accuracy in cognitive tasks) based on self-reported anxiety.

Accuracy									
Algorithm	Accuracy	F1-Score	ROC AUC	Accuracy	F1-Score	ROC AUC	Accuracy	F1-Score	ROC AUC
	Model 1			Model 2			Model 3		
	Numeric task								
KNN	0.430	0.437	0.460	0.500	0.574	0.447	0.430	0.462	0.425
Linear SVC	0.605	0.630	N/A	0.442	0.400	N/A	0.477	0.505	N/A
Random Forest	0.465	0.549	0.425	0.442	0.478	0.377	0.465	0.540	0.390
Decision Tree	0.465	0.549	0.420	0.430	0.542	0.378	0.430	0.542	0.378
	Spatial task								
KNN	0.651	0.623	0.643	0.651	0.667	0.688	0.663	0.696	0.699
Linear SVC	0.627	0.627	N/A	0.566	0.571	N/A	0.566	0.600	N/A
Random Forest	0.639	0.652	0.685	0.663	0.682	0.698	0.627	0.635	0.689
Decision Tree	0.651	0.701	0.690	0.651	0.667	0.615	0.675	0.697	0.650
	Social task								
KNN	0.553	0.575	0.582	0.566	0.601	0.621	0.623	0.655	0.651
Linear SVC	0.535	0.532	N/A	0.535	0.513	N/A	0.535	0.565	N/A
Random Forest	0.541	0.510	0.625	0.585	0.616	0.625	0.597	0.614	0.646
Decision Tree	0.547	0.514	0.624	0.572	0.564	0.629	0.572	0.564	0.634
	Control task								
KNN	0.514	0.526	0.570	0.608	0.695	0.654	0.500	0.565	0.585
Linear SVC	0.432	0.447	N/A	0.459	0.459	N/A	0.514	0.571	N/A
Random Forest	0.635	0.649	0.729	0.649	0.658	0.708	0.635	0.658	0.725
Decision Tree	0.622	0.588	0.673	0.662	0.638	0.707	0.689	0.635	0.734
Reaction time									
Algorithm	MAE	RMSE	R^2^	MAE	RMSE	R^2^	MAE	RMSE	R^2^
	Model 1			Model 2			Model 3		
	Numeric task								
KNN	0.131	0.221	0.005	0.128	0.215	0.056	0.128	0.214	0.064
SVR	0.123	0.218	0.028	0.121	0.216	0.048	0.108	0.201	0.179
Random Forest	0.130	0.214	0.064	0.118	0.203	0.161	0.117	0.202	0.171
Decision Tree	0.130	0.214	0.065	0.117	0.202	0.169	0.117	0.202	0.169
	Social task								
KNN	0.309	0.526	−1.577	0.189	0.356	−0.179	0.179	0.318	0.055
SVR	0.169	0.327	0.000	0.166	0.325	0.013	0.149	0.304	0.138
Random Forest	0.180	0.313	0.083	0.162	0.303	0.143	0.162	0.303	0.144
Decision Tree	0.180	0.313	0.084	0.162	0.303	0.141	0.162	0.303	0.140
	Spatial task								
KNN	0.493	0.759	−0.193	0.437	0.708	−0.039	0.424	0.698	−0.009
SVR	0.379	0.714	−0.054	0.372	0.703	−0.021	0.354	0.678	0.048
Random Forest	0.410	0.669	0.074	0.393	0.649	0.128	0.394	0.649	0.127
Decision Tree	0.409	0.668	0.074	0.393	0.649	0.128	0.393	0.649	0.127
	Color task								
KNN	0.204	0.308	0.150	0.204	0.313	0.123	0.209	0.315	0.112
SVR	0.195	0.310	0.137	0.206	0.322	0.067	0.187	0.302	0.182
Random Forest	0.196	0.299	0.199	0.196	0.299	0.199	0.195	0.298	0.200
Decision Tree	0.195	0.298	0.200	0.196	0.299	0.200	0.195	0.299	0.199

Note: For Accuracy: Model 1—performance is predicted only from domain-specific anxieties; Model 2—performance is predicted from general anxiety (trait/state/generalized anxiety); Model 3—performance is predicted from all anxiety measures; Accuracy is the proportion of true positive and true negative results; F1-score is the mean of precision and recall; AUC, the receiver operating characteristic area under the curve, measures the ability of the model to distinguish between classes. For Reaction time: Model 1—performance is predicted from domain-specific anxieties; Model 2—performance is predicted from general anxiety (trait/state/generalized anxiety); Model 3—performance is predicted from all anxiety measures; MAE (Mean Absolute Error)—in seconds; RMSE (Root Mean Square Error)—a quadratic scoring rule that measures the average magnitude of the errors; R^2^—indicates the proportion of variance.

## Data Availability

The data presented in this study are openly available in OSF Repository at https://osf.io/2q74n/overview, accessed on 25 March 2026.

## Supplementary Materials

The following supporting information can be downloaded at: https://www.mdpi.com/article/10.3390/bs16050806/s1, Table S1: Descriptives of anxiety questionnaires for Study 1; Figure S1: Correlations of anxiety questionnaires; Figure S2a: Correlations of anxiety questionnaires and correct responses; Figure S2b: Correlations of anxiety questionnaires and reaction time; Figure S3a: Correct Responses and Color Task; Figure S3b: Correct Responses and Social Task; Figure S3c: Correct Responses and Spatial Task; Figure S3d: Correct Responses and Numeric Task; Figure S4a: Reaction Time and Colour Task; Figure S4b: Reaction Time and Social Task; Figure S4c: Reaction Time and Spatial Task; Figure S4d: Reaction Time and Numeric Task; Table S2a: Exploratory Factor Loadings by items; Table S2b: Confirmatory Factor Loading by items.
