# Supplementary material for: General and Specific Facets of Anxiety: Psychometric Analysis and Impact on Cognitive Performance
Source: Behav Sci (Basel). 2026 May 18;16(5):806. doi: 10.3390/bs16050806 (PMC13203332; doi:10.3390/bs16050806)
Supplement: Supplementary file 1 [file behavsci-16-00806-s001.zip › behavsci-4252088-supplementary.pdf]

Supplementary Materials for General and Specific Facets of Anxiety: Psychometric Analysis and Impact on Cognitive Performance

Table S1 Descriptives of anxiety questionnaires for Study 1

	Mean	SD	Skewness	Kurtosis	Chronbach's alpha
**Trait Anxiety**	43.98	10.59	.16	-.38	.90
**State Anxiety**	39.26	11.12	.60	-.10	.91
**Generalized Anxiety**	6.50	4.09	.69	.25	.83
**Maths Anxiety**	18.93	6.46	.68	.00	.86
**Social Anxiety**	2.39	0.82	.29	-.69	.93
**Spatial Anxiety**	22	7.46	.17	-.76	.86

Notes: Trait Anxiety - State Trait Anxiety Inventory (Trait subscale); State Anxiety - State Trait Anxiety Inventory (State Subscale); Generalised Anxiety - Generalised Anxiety Disorder Questionnaire; Maths Anxiety - The Abbreviated Math Anxiety Scale; Social Anxiety - Appraisal of Social Concerns; Spatial Anxiety - Spatial Anxiety Questionnaire;

Figure S1 Correlations of anxiety questionnaires

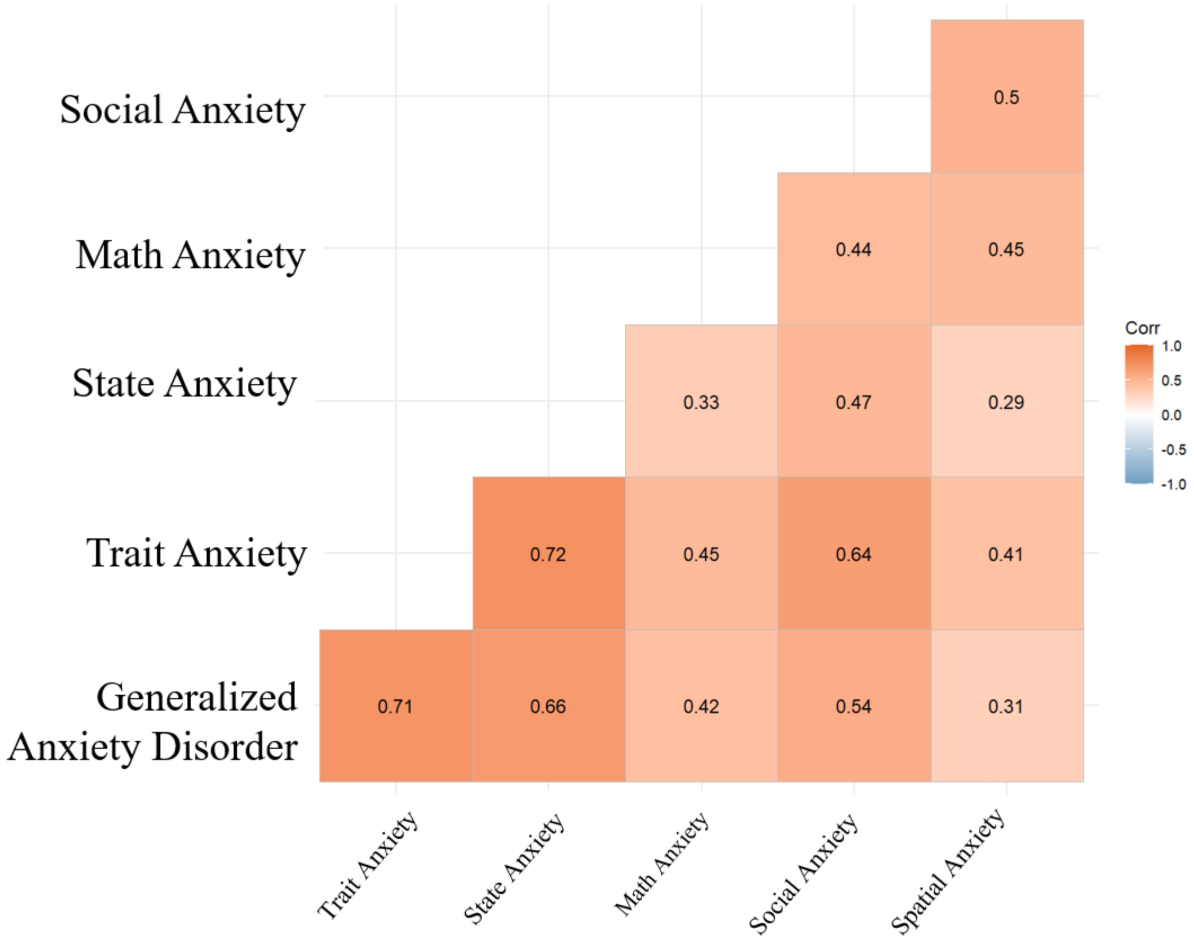

Notes: all present correlations p< .05; Insignificant correlations are crossed out; Trait Anxiety - State-Trait Anxiety Inventory Trait Subscale; State Anxiety - State-Trait Anxiety Inventory State Subscale; Generalised Anxiety Disorder - Generalised Anxiety Disorder Questionnaire; Social Anxiety - Appraisal of Social Concerns; Spatial Anxiety - Spatial Anxiety Questionnaire; Maths Anxiety - Abbreviated Maths Anxiety Scale;

Figure S2a Correlations of anxiety questionnaires and correct responses

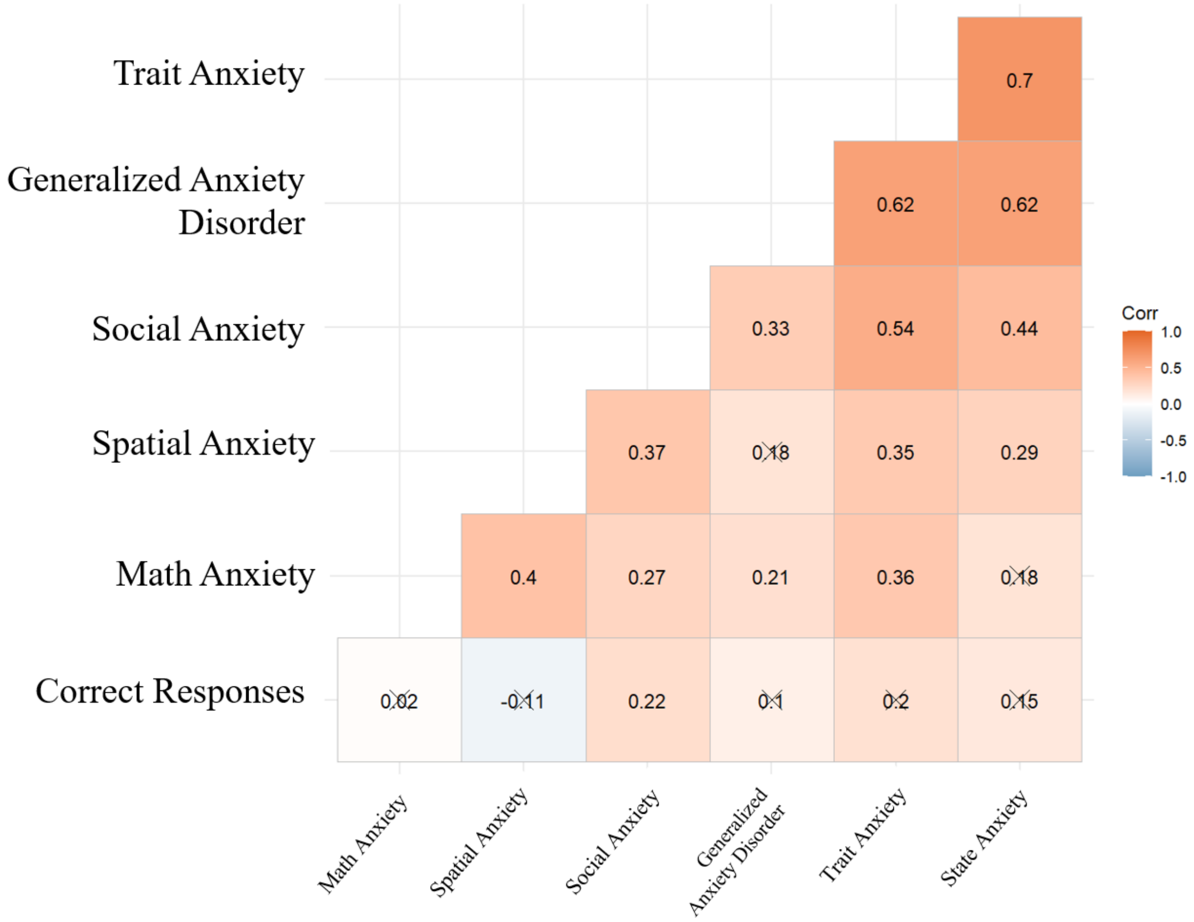

Notes: all present correlations p< .05; Insignificant correlations are crossed out; Trait Anxiety - State-Trait Anxiety Inventory Trait Subscale; State Anxiety - State-Trait Anxiety Inventory State Subscale; Generalised Anxiety Disorder - Generalised Anxiety Disorder Questionnaire; Social Anxiety - Appraisal of Social Concerns; Spatial Anxiety - Spatial Anxiety Questionnaire; Maths Anxiety - Abbreviated Maths Anxiety Scale; Correct Responses - sum of correct responses;

Figure S2b Correlations of anxiety questionnaires and reaction time

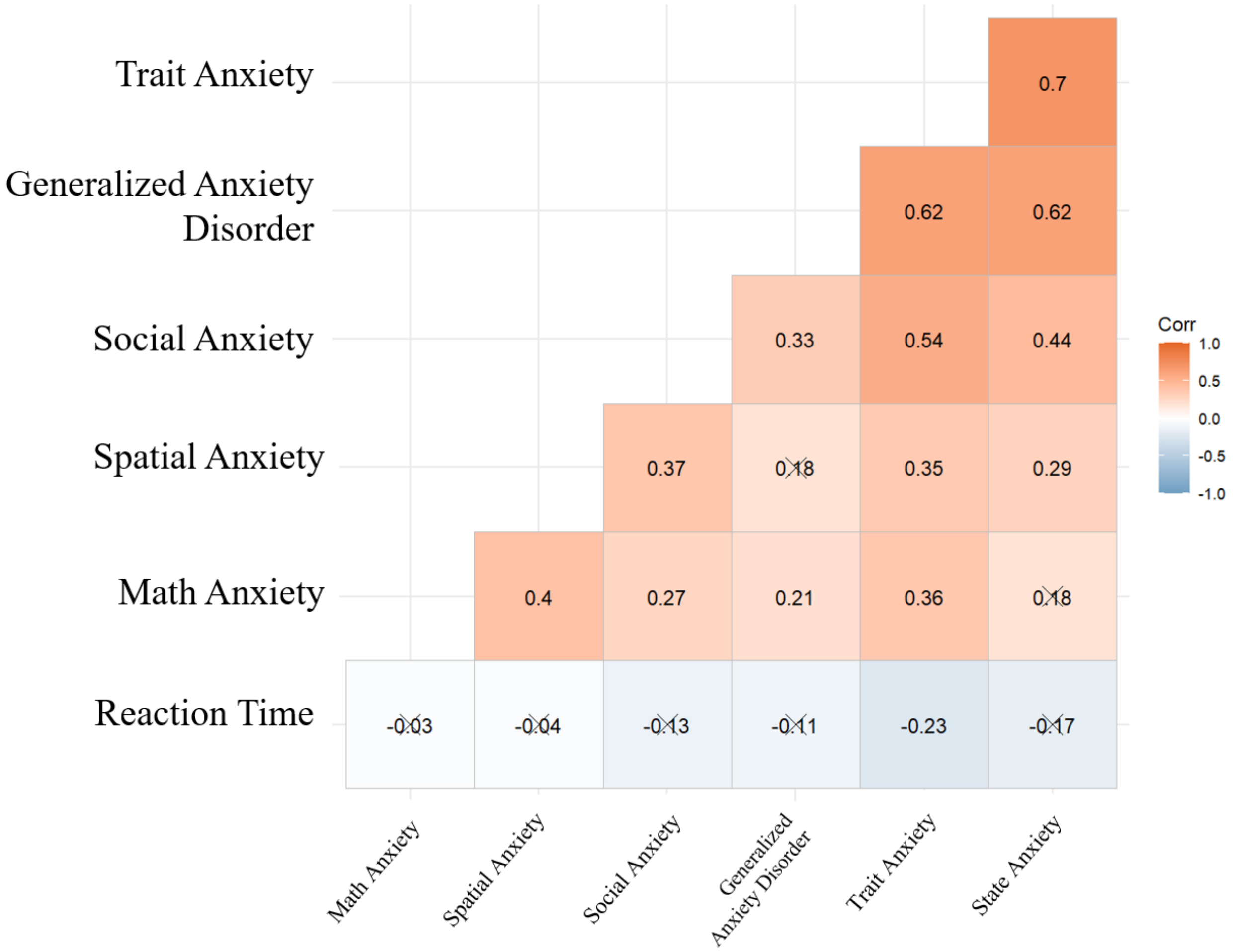

Notes: all present correlations p< .05; Insignificant correlations are crossed out; all present correlations p< .05; Insignificant correlations are crossed out; Trait Anxiety - State-Trait Anxiety Inventory Trait Subscale; State Anxiety - State-Trait Anxiety Inventory State Subscale; Generalised Anxiety Disorder - Generalised Anxiety Disorder Questionnaire; Social Anxiety - Appraisal of Social Concerns; Spatial Anxiety - Spatial Anxiety Questionnaire; Maths Anxiety - Abbreviated Maths Anxiety Scale; Reaction Time - mean of all reaction times;

Figure S3a Correct Responses and Colour Task

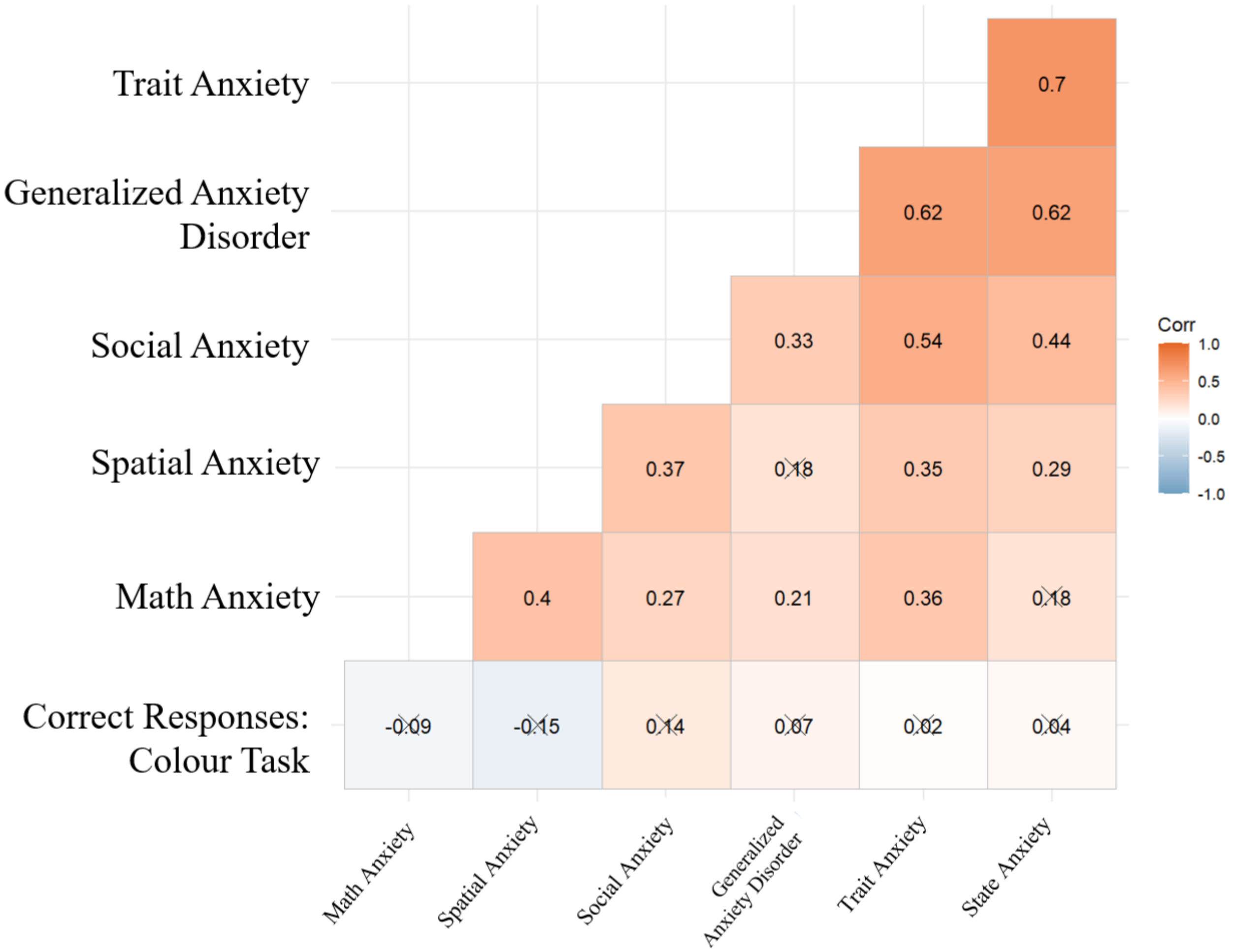

Notes: Trait Anxiety - State-Trait Anxiety Inventory; Generalised Anxiety Disorder - Generalised Anxiety Disorder Questionnaire; Social Anxiety - Appraisal of Social Concerns; Spatial Anxiety - Spatial Anxiety Questionnaire; Maths Anxiety - Abbreviated Maths Anxiety Scale; Correct Responses: Colour Task - sum of correct responses for Colour Stroop Task;

Figure S3b Correct Responses and Social Task

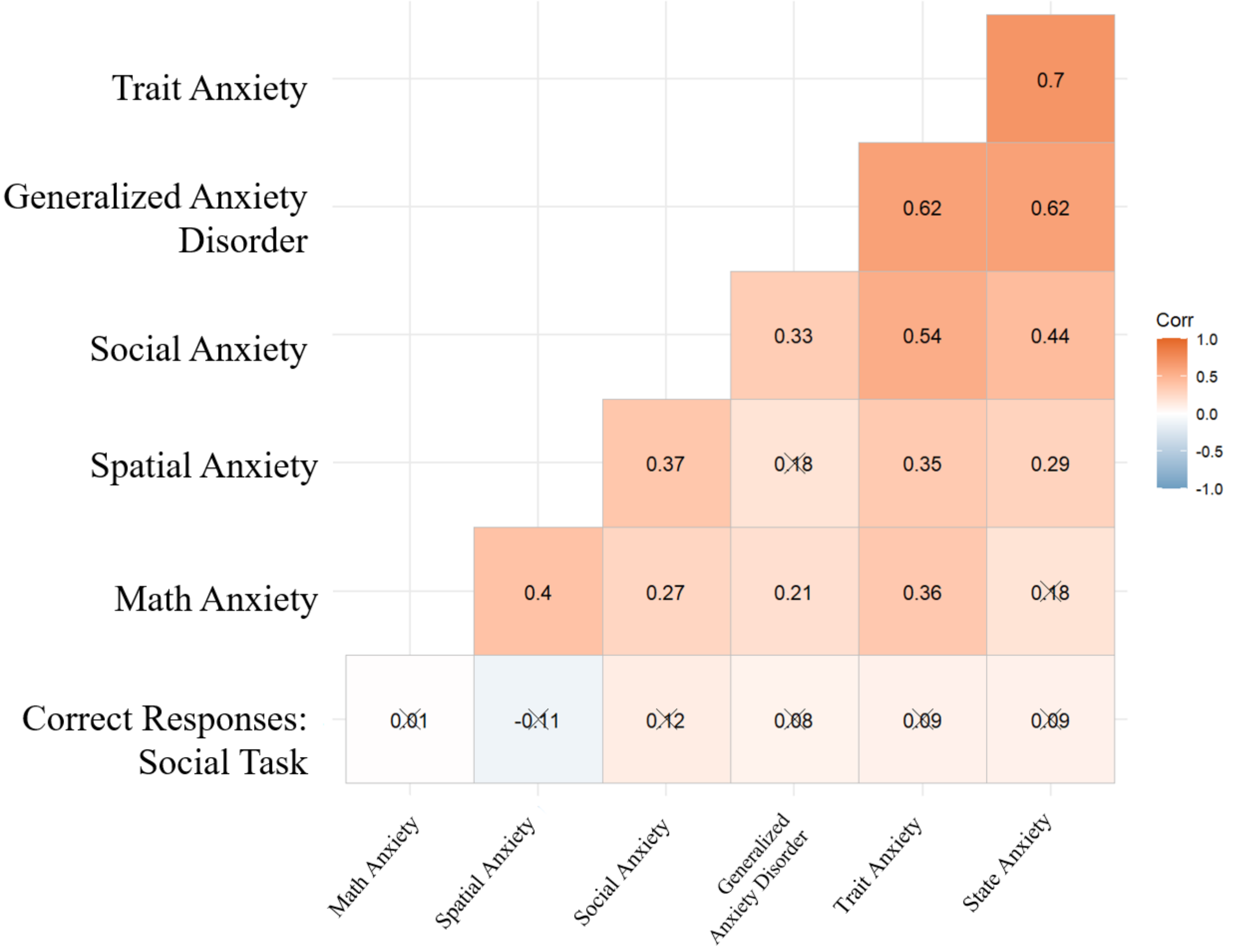

Notes: Trait Anxiety - State-Trait Anxiety Inventory; Generalised Anxiety Disorder - Generalised Anxiety Disorder Questionnaire; Social Anxiety - Appraisal of Social Concerns; Spatial Anxiety - Spatial Anxiety Questionnaire; Maths Anxiety - Abbreviated Maths Anxiety Scale; Correct Responses: Social Task - sum of correct responses for Social Stroop Task;

Figure S3c Correct Responses and Spatial Task

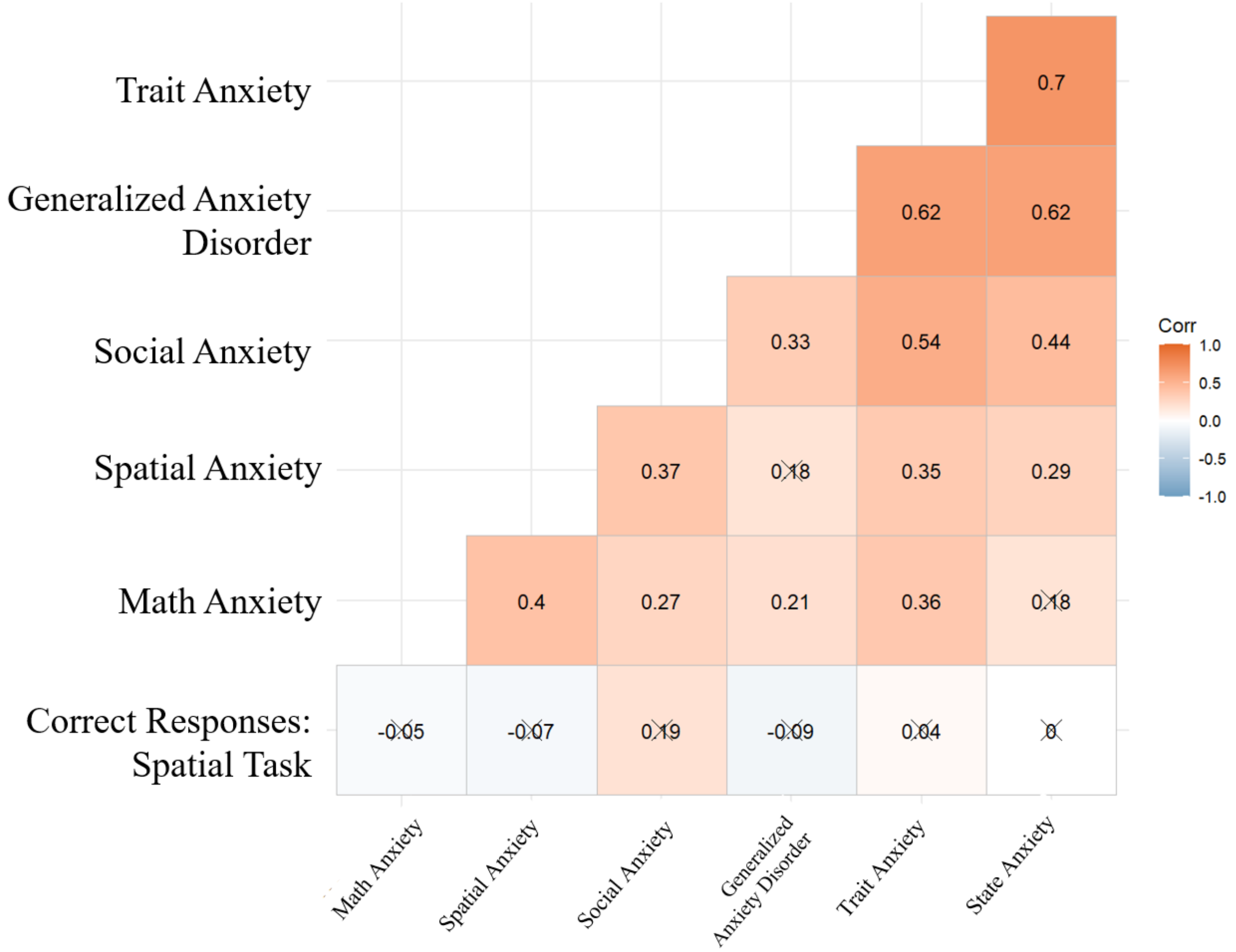

Notes: Trait Anxiety - State-Trait Anxiety Inventory; Generalised Anxiety Disorder - Generalised Anxiety Disorder Questionnaire; Social Anxiety - Appraisal of Social Concerns; Spatial Anxiety - Spatial Anxiety Questionnaire; Maths Anxiety - Abbreviated Maths Anxiety Scale; Correct Responses: Spatial Task - sum of correct responses for Flanker Task;

Figure S3d Correct Responses and Numeric Task

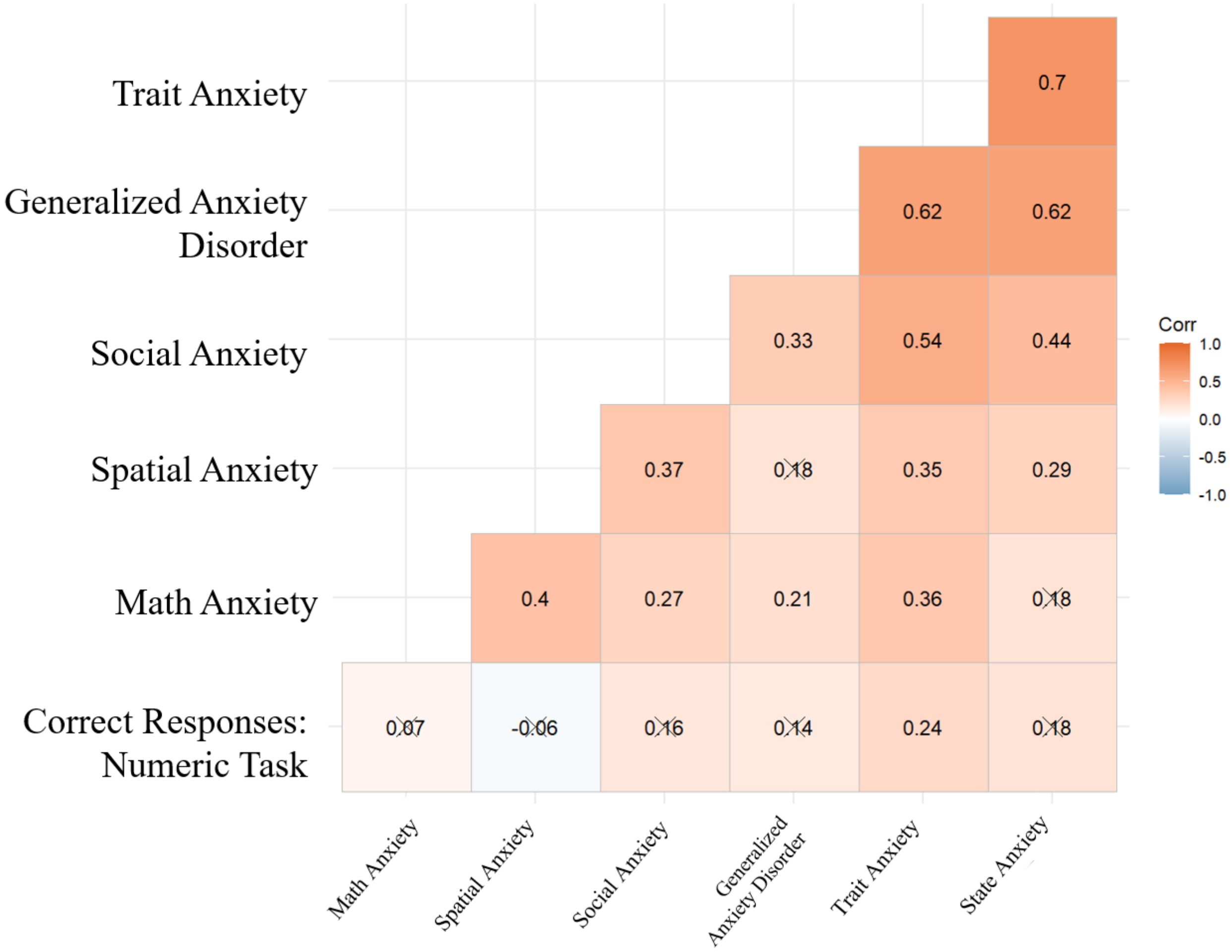

Notes: Trait Anxiety - State-Trait Anxiety Inventory; Generalised Anxiety Disorder - Generalised Anxiety Disorder Questionnaire; Social Anxiety - Appraisal of Social Concerns; Spatial Anxiety - Spatial Anxiety Questionnaire; Maths Anxiety - Abbreviated Maths Anxiety Scale; Correct Responses: NumericTask - sum of correct responses for Numeric Stroop Task;

Figure S4a Reaction Time and Colour Task

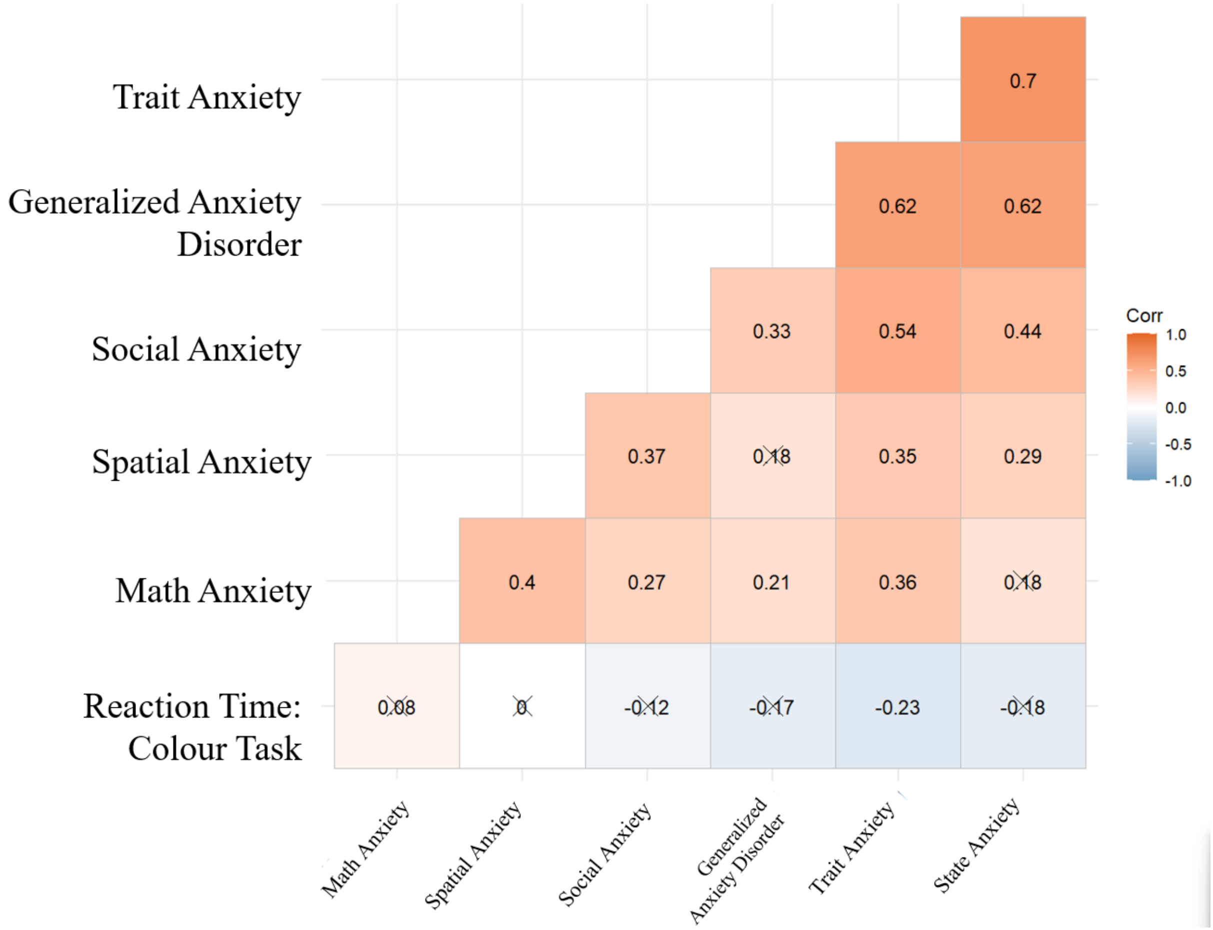

Notes: Trait Anxiety - State-Trait Anxiety Inventory; Generalised Anxiety Disorder - Generalised Anxiety Disorder Questionnaire; Social Anxiety - Appraisal of Social Concerns; Spatial Anxiety - Spatial Anxiety Questionnaire; Maths Anxiety - Abbreviated Maths Anxiety Scale; Reaction Time: Colour Task - mean reaction time for Colour Stroop Task;

Figure S4b Reaction Time and Social Task

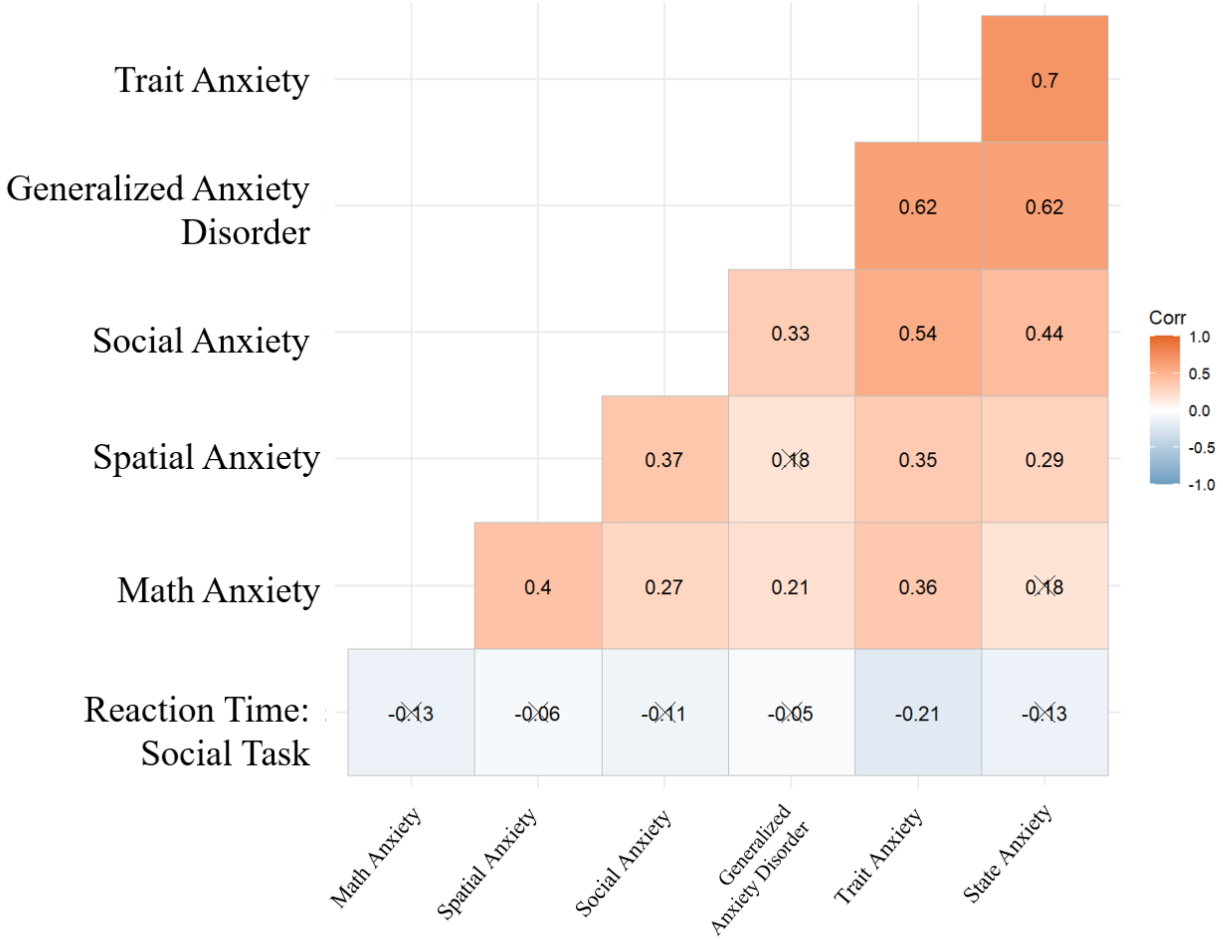

Notes: Trait Anxiety - State-Trait Anxiety Inventory; Generalised Anxiety Disorder - Generalised Anxiety Disorder Questionnaire; Social Anxiety - Appraisal of Social Concerns; Spatial Anxiety - Spatial Anxiety Questionnaire; Maths Anxiety - Abbreviated Maths Anxiety Scale; Reaction Time: Social Task - mean reaction time for Social Stroop Task;

Figure S4c Reaction Time and Spatial Task

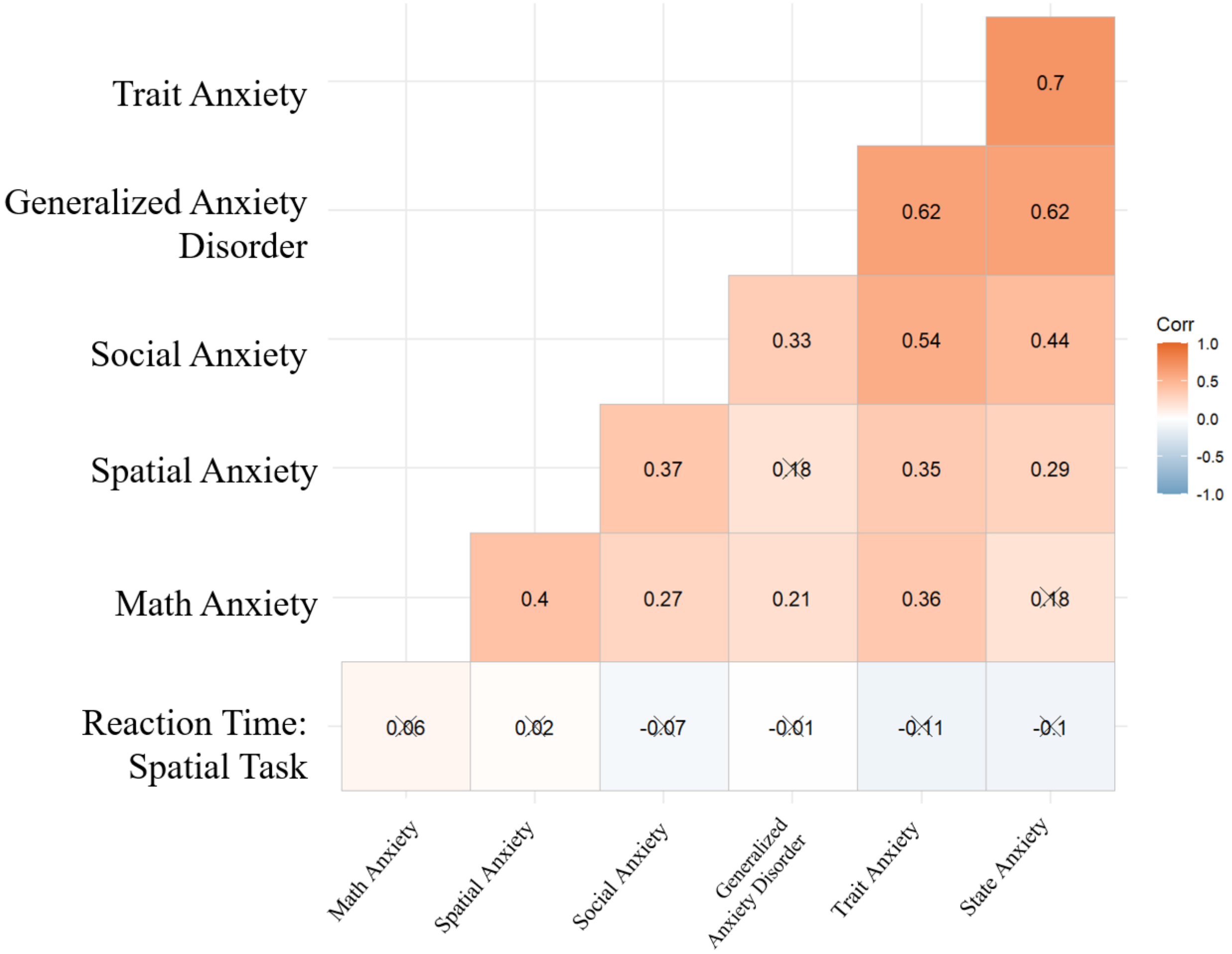

Notes: Trait Anxiety - State-Trait Anxiety Inventory; Generalised Anxiety Disorder - Generalised Anxiety Disorder Questionnaire; Social Anxiety - Appraisal of Social Concerns; Spatial Anxiety - Spatial Anxiety Questionnaire; Maths Anxiety - Abbreviated Maths Anxiety Scale; Reaction Time: Social Task - mean reaction time for Social Stroop Task;

Figure S4d Reaction Time and Numeric Task

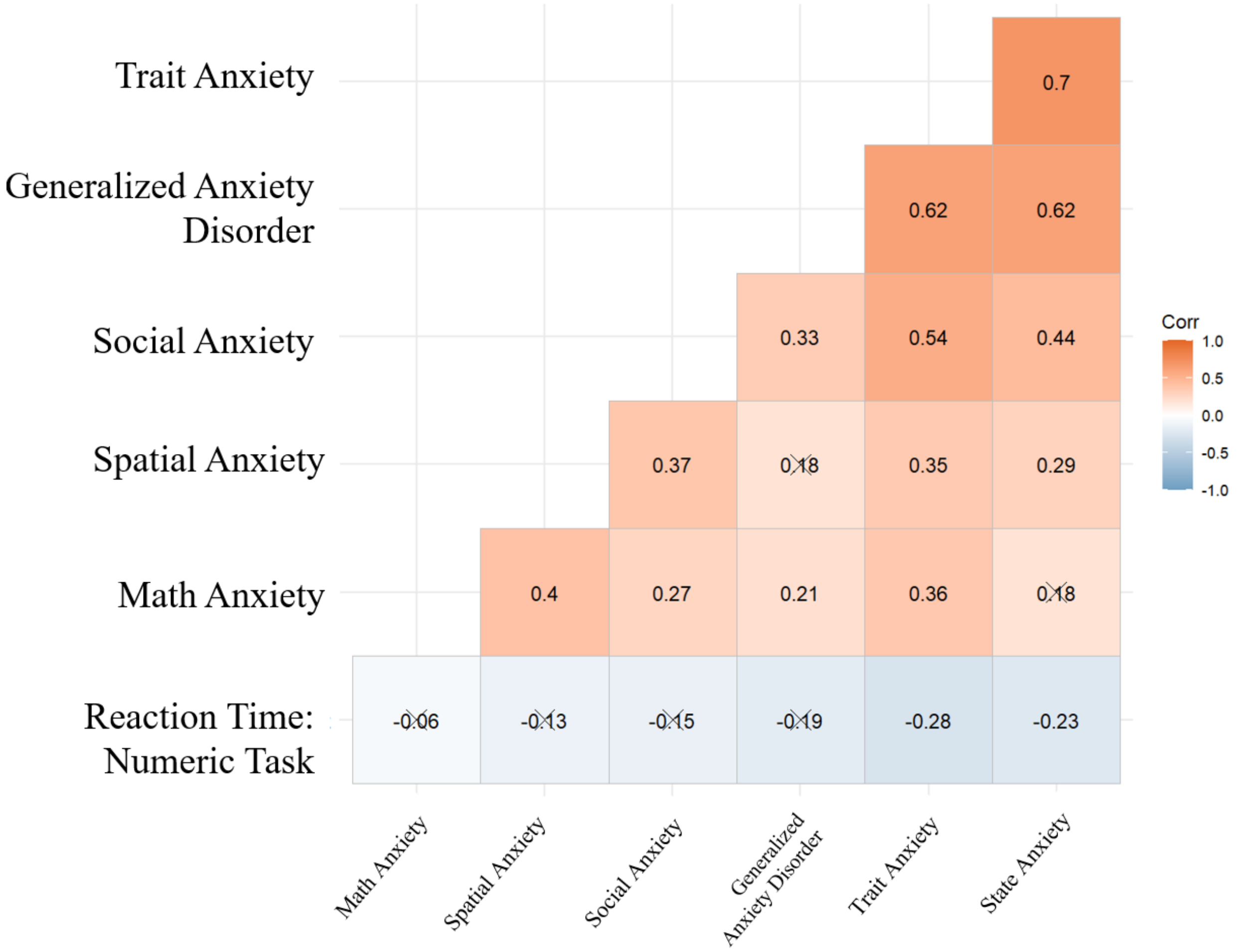

Notes: Trait Anxiety - State-Trait Anxiety Inventory; Generalised Anxiety Disorder - Generalised Anxiety Disorder Questionnaire; Social Anxiety - Appraisal of Social Concerns; Spatial Anxiety - Spatial Anxiety Questionnaire; Maths Anxiety - Abbreviated Maths Anxiety Scale; Reaction Time: Numeric Task - mean reaction time for Numeric Stroop Task;

Table S2a Exploratory Factor Loadings by items

Item	1	2	3	4	5	6	7	8	9	10	11	12	13	14	15	16	17	Communality
STAIS1	.06	.08	.12	.03	.02	.12	.29	.02	-.02	-.07	.01	.04	.16	-.04	.08	**.29**	-.02	**.55**
STAIS2	.03	-.03	-.00	-.05	-.02	.04	**.82**	.06	-.03	.03	.06	-.02	.02	.00	.05	-.01	-.00	**.74**
STAIS3	-.01	-.09	.04	.12	.05	.16	.15	.18	.02	-.05	.08	-.08	.10	.12	.02	**.30**	.00	**.49**
STAIS4	.00	-.02	-.00	-.00	.04	-.02	.01	**.83**	.04	-.04	.01	.03	-.02	-.02	.01	.03	.00	**.71**
STAIS5	-.04	.06	-.19	.11	.04	.03	.23	.07	.12	.11	-.02	.01	.12	-.11	.01	.01	.17	.28
STAIS6	.04	-.01	-.02	.04	-.04	.11	.04	**.67**	.00	.06	-.07	-.01	.02	-.02	.04	.04	-.00	**.59**
STAIS7	-.09	.06	.08	.10	-.02	.04	-.06	.22	.06	.08	.07	.03	.01	.20	.03	**.43**	-.01	**.53**
STAIS8	.05	-.02	.01	.06	.04	.00	.05	.04	.01	.09	.03	-.05	**.65**	-.04	.00	.02	.02	**.57**
STAIS9	.09	-.08	.02	.04	.06	**.33**	.09	.17	.00	.03	.02	-.01	.10	.09	-.06	**.39**	.04	**.64**
STAIS10	.03	.21	.03	.05	.03	-.03	**.32**	.11	.03	.01	-.01	.00	.24	-.02	.01	.18	-.01	**.60**
STAIS11	-.01	**.37**	.12	.06	-.01	-.09	.16	.08	.05	.17	-.01	-.04	.03	.05	.06	.16	.18	**.59**
STAIS12	.03	-.02	.10	.05	.04	**.35**	.06	.13	-.01	.04	.04	.00	.03	-.01	-.00	**.45**	.00	**.66**
STAIS13	-.04	-.02	.14	.06	-.00	**.43**	.07	.16	.00	.02	.01	-.02	.09	-.00	.07	.17	-.03	**.54**
STAIS14	-.00	.02	-.00	.08	-.05	**.66**	.05	-.06	.07	-.05	.01	.06	-.00	.04	.01	.04	.04	**.54**
STAIS15	.00	.06	.06	.01	-.02	.13	.28	-.04	-.00	-.09	.00	.17	**.42**	.02	.00	.21	.01	**.66**
STAIS16	-.00	**.30**	-.09	.05	.08	-.08	.24	.18	.00	-.03	.00	.01	.28	.06	-.01	.05	.02	**.61**
STAIS17	-.00	-.01	.03	-.00	.04	**.54**	.01	.12	-.03	-.04	.06	.05	.07	.02	-.02	.24	.03	**.57**
STAIS18	.06	.01	.00	.05	-.00	**.69**	.02	.08	.05	.03	-.05	-.02	-.01	-.08	.03	-.06	-.05	**.59**
STAIS19	.03	.29	-.00	.02	.01	-.21	.05	.15	.01	-.06	-.05	-.02	**.34**	.20	.03	-.07	-.03	**.49**
STAIS20	-.02	**.29**	-.04	.09	.05	.02	.13	.20	.06	.02	-.01	.07	**.30**	.03	.02	.07	-.09	**.62**
STAIT1	-.07	**.47**	.01	.01	.02	.01	.14	.11	.00	-.01	.02	.04	.19	.04	.11	-.08	.01	**.58**
STAIT2	.08	**.35**	.05	.19	.00	.08	-.01	.02	-.08	.06	.15	-.06	.01	-.06	.06	-.03	.26	**.45**
STAIT3	.09	.09	.24	.11	.06	.00	.04	.17	-.02	-.02	.07	-.01	.03	-.02	.02	-.13	.05	.31
STAIT4	.02	.27	.11	-.01	-.09	.13	-.00	**.30**	-.01	.02	.06	.18	-.07	.04	.00	-.15	-.04	.37
STAIT5	.07	**.31**	.07	-.03	.05	.06	-.00	.15	-.00	.04	.09	.01	-.29	.09	.00	.08	.15	.33
STAIT6	.01	**.31**	.08	.12	.00	.11	-.02	-.03	-.05	.03	.11	-.04	**.47**	.01	.05	-.05	.08	**.60**
STAIT7	.10	**.46**	.26	.03	.05	.00	.09	-.10	.02	.02	.01	.06	.05	-.10	-.03	.15	.12	**.57**
STAIT8	.03	.24	.06	.22	.09	.10	.00	.11	.04	.10	-.06	-.03	-.12	.00	-.00	.13	.13	.36
STAIT9	.01	.06	**.58**	-.03	.09	.04	.05	.02	-.04	.05	.07	.03	.00	.01	.03	.08	-.03	**.56**
STAIT10	.04	**.51**	.08	.01	-.03	-.06	.17	.12	-.01	.03	-.05	.00	.22	.05	-.02	-.08	.00	**.67**
STAIT11	.07	-.05	**.58**	.15	.02	-.05	-.05	-.03	.02	.00	.05	.02	.03	-.05	.01	.01	.02	**.46**
STAIT12	.04	.24	**.34**	.01	-.01	-.01	-.00	.09	.07	.16	.03	-.02	-.02	.14	-.02	-.04	.18	**.53**
STAIT13	.00	.12	.05	.10	.08	-.03	**.65**	-.04	.09	.01	-.04	.06	-.03	.02	.00	-.02	-.00	**.65**
STAIT14	.03	-.02	.17	-.20	.02	-.01	-.10	.11	.06	.06	.07	-.11	.04	.12	-.03	-.06	**.30**	.21
STAIT15	-.03	.21	.14	.23	.00	-.07	-.03	**.32**	.04	-.05	-.02	.11	.04	.07	.08	-.15	.06	**.50**
STAIT16	.03	**.60**	-.08	.12	.03	.04	.12	.06	.03	-.01	-.01	.11	.08	.06	.03	.01	-.05	**.69**
STAIT17	-.04	-.02	**.58**	.11	.02	.00	.05	-.03	.11	-.01	-.06	.08	.01	.06	.00	.08	-.02	**.52**
STAIT18	.07	.04	**.46**	.04	.06	.01	.02	.16	.08	-.04	.00	.10	.05	.06	.02	-.09	-.05	**.52**

STAIT 19	.14	**.39**	.16	.15	.06	-.05	.11	-.01	.06	.07	.02	.02	-.05	-.09	.01	.01	.09	**.53**
STAIT 20	.03	-.06	**.44**	.10	.07	.19	.17	.02	.00	-.04	.07	.03	-.03	.05	.04	-.10	.09	**.55**
GAD1	.06	.02	.02	**.62**	.03	-.00	-.07	.06	.00	.02	.10	.05	.15	.03	-.06	.04	.02	**.65**
GAD2	-.06	.05	.09	**.63**	.12	.11	.00	.02	-.02	.02	.05	.05	-.02	-.03	.05	.01	-.03	**.67**
GAD3	.01	.00	.18	**.51**	-.03	-.03	.09	.02	.00	.01	.10	.08	-.02	.03	.04	.01	-.02	**.56**
GAD4	.00	-.04	-.01	**.44**	.01	.05	.16	.02	.06	.08	.08	-.01	.12	-.05	.03	.05	.19	**.53**
GAD5	-.02	-.01	.12	**.38**	.02	.26	.12	.09	.04	-.01	-.06	-.03	-.07	.06	.01	-.18	-.06	**.44**
GAD6	.07	.02	.07	.26	.06	.03	-.04	.01	.09	-.02	-.02	.06	.14	-.01	.06	.05	.05	.27
GAD7	-.00	-.12	.18	.17	-.02	.18	.17	.02	.21	.00	-.03	.09	-.01	.08	.03	-.13	.00	.37
AMAS 1	-.01	.03	.16	-.07	.08	.23	.02	-.00	.25	.13	.21	-.09	.01	-.12	.09	-.10	**-.31**	**.48**
AMAS 2	.05	.06	.04	.00	.01	.01	-.04	.00	.08	.07	**.64**	.01	-.01	.07	.02	.06	-.15	**.63**
AMAS 3	-.02	.09	.01	-.02	.11	.04	.04	.01	**.45**	-.04	.26	.03	-.08	-.05	.04	.01	-.10	**.49**
AMAS 4	.04	.00	-.01	.03	.02	.09	-.03	-.01	.08	.02	**.69**	-.00	.01	-.02	-.02	-.04	-.00	**.58**
AMAS 5	.02	.01	.05	.07	-.00	-.04	.05	-.02	**.35**	-.01	**.42**	-.00	-.05	.07	.00	.04	-.02	**.53**
AMAS 6	.04	-.03	-.04	.03	-.03	-.01	-.00	.02	**.85**	.00	.01	-.00	.04	-.02	-.00	-.02	.00	**.72**
AMAS 7	-.00	-.01	.01	-.01	.07	-.01	-.01	.04	**.62**	-.04	.00	.05	-.03	.07	.03	.01	-.00	**.46**
AMAS 8	.04	-.07	-.01	.05	.02	-.09	.08	-.01	.01	-.04	**.77**	.06	.03	.02	-.00	-.01	.10	**.71**
AMAS 9	.01	.03	.10	-.11	-.02	.09	-.00	-.00	**.61**	.04	.13	-.03	-.02	-.06	.09	.06	.03	**.58**
ASC1	.02	.00	.05	.04	**.78**	.03	-.00	.01	-.05	-.02	.04	-.02	.00	.02	-.03	.01	-.14	**.63**
ASC2	.07	.10	.06	.02	.03	-.07	.10	.00	-.00	**.46**	.07	-.05	-.04	.51	-.00	.03	-.11	**.78**
ASC3	.03	.01	-.00	.04	.02	-.02	.02	-.02	.04	**.77**	.04	.11	-.00	.04	.01	.04	-.00	**.82**
ASC4	.08	-.06	.03	.00	.24	-.05	.01	.15	.03	.26	.07	-.07	-.02	.03	.08	-.12	-.03	.30
ASC5	.10	.09	.02	.04	.02	-.01	.00	.00	.10	.05	-.01	**.52**	-.04	.08	.05	-.02	-.01	**.50**
ASC6	.07	.02	.10	-.02	.25	.01	.02	-.07	.10	**.31**	-.02	.06	.06	.04	.00	-.03	.25	**.54**
ASC7	.00	-.05	-.00	.03	**.78**	-.09	.05	.02	.05	.04	-.01	.01	-.00	-.09	.02	.00	.07	**.66**
ASC8	.02	.12	-.01	-.09	**.52**	.02	-.05	-.02	.05	-.00	.09	.04	.03	.22	.06	.01	.12	**.59**
ASC9	.06	-.02	.05	.04	.02	-.02	-.00	-.02	-.06	.08	.14	.13	.03	**.58**	.06	.10	.06	**.69**
ASC10	.06	-.00	.06	-.04	**.31**	.04	.05	.03	.01	-.07	.05	.08	-.04	**.46**	.04	-.02	.17	**.63**
ASC11	-.01	-.01	.02	-.05	**.29**	.10	-.03	-.07	.03	.19	-.08	.20	.14	-.06	.08	-.01	.08	.33
ASC12	.11	-.07	.18	.00	.02	-.00	-.01	.04	-.05	.08	.09	.16	.10	**.39**	.05	.01	.18	**.62**
ASC13	.10	.03	-.05	.14	.31	.15	-.01	-.07	.12	-.06	-.09	.12	-.00	.21	.05	-.04	.12	**.43**
ASC14	.11	-.01	-.05	.14	.07	.15	.00	.01	.11	.27	-.07	.12	-.00	.26	-.00	-.15	.12	**.52**
ASC15	-.00	-.05	.03	.00	.07	.02	.03	.04	-.03	**.62**	.00	.28	.09	-.02	.05	-.04	.09	**.80**
ASC16	-.02	.07	-.03	.08	.11	.12	-.02	-.01	.04	.14	.08	.03	.02	.25	.15	-.03	**.32**	**.52**
ASC17	.00	-.04	.07	-.04	.05	.15	-.02	.04	.03	.27	.03	**.33**	.14	.10	.05	-.24	.00	**.56**
ASC18	.01	.01	.01	.05	.09	.12	-.00	.04	.07	.19	-.02	**.42**	-.01	.12	-.01	-.03	-.04	**.50**
ASC19	.03	.01	.06	.05	.00	-.03	.02	.05	-.02	.14	.10	**.70**	-.04	-.03	.02	.03	.00	**.75**
ASC20	.09	-.10	.07	-.00	**.29**	.08	-.05	.09	-.00	.12	.06	.16	.04	-.01	.03	-.05	.05	.36
SA1	**.71**	.02	.00	.02	.02	.05	.03	.04	-.04	.06	.07	-.04	.02	-.01	.00	-.13	-.05	**.60**
SA2	**.39**	-.02	.01	.01	-.00	.02	-.09	.02	.09	.05	-.03	.07	.05	.11	.13	.02	-.06	.34
SA3	**.60**	-.03	.06	.06	.04	-.00	-.08	.01	-.00	.05	-.02	-.03	.06	.02	.12	.06	-.11	**.50**
SA4	**.33**	.05	.02	-.04	.05	.07	-.06	.05	.11	.03	-.01	-.07	-.06	-.02	.22	.00	-.12	.30
SA5	**.83**	-.03	.04	-.02	-.01	-.03	.05	.02	.06	-.04	-.02	.05	.00	-.01	-.05	.00	.04	**.71**
SA6	**.68**	.02	-.08	-.07	.00	.04	.01	-.04	.01	-.00	.06	.00	-.03	-.00	.06	.03	.05	**.54**
SA7	**.54**	.07	-.07	.02	.04	-.07	-.06	-.01	-.03	-.01	.09	.05	-.00	.07	.06	.09	.05	**.44**
SA8	.11	-.03	-.00	-.00	-.03	-.00	.03	.01	.04	-.09	-.03	.07	-.01	.05	**.67**	.00	.00	**.56**

SA9	-.02	-.00	-.01	-.01	-.00	-.02	-.00	-.00	-.00	.01	-.01	-.03	.00	-.02	**.01**	-.00	-.00	**.98**
SA10	**.41**	-.00	.01	.06	.02	.05	.01	-.14	.01	-.05	.04	.07	-.08	.03	.23	.06	-.04	**.40**

Notes: STAIS - items of State Trait Anxiety Inventory State subscale; STAIT - items of State Trait Anxiety Inventory Trait Subscale; GAD - items of Generalised Anxiety Disorder Questionnaire; AMAS - items of Abbreviated Maths Anxiety Scale; ASC - items of Appraisal of Social concerns; SA - items of Social Anxiety Questionnaire; for factor loadings, values > **0.3** are highlighted in **bold**; for communality, values > **0.4** are highlighted in **bold**;

Table S2b Confirmatory Factor Loading by items.

Latent Variable	Item	Factor Loading	Communality
Trait	STAIT1	.64	.41
	STAIT2	.59	.34
	STAIT3	.51	.26
	STAIT4	.49	.24
	STAIT5	.41	.17
	STAIT6	.65	.42
	STAIT7	.68	.46
	STAIT8	.53	.29
	STAIT9	.61	.37
	STAIT10	.66	.43
	STAIT11	.48	.23
	STAIT12	.63	.40
	STAIT13	.64	.41
	STAIT14	.12	.01
	STAIT15	.62	.38
	STAIT16	.70	.50
	STAIT17	.55	.30
	STAIT18	.63	.40
	STAIT19	.67	.45
	STAIT20	.60	.36

State	STAIS1	.69	.48
	STAIS2	.63	.40
	STAIS3	.62	.39
	STAIS4	.54	.29
	STAIS5	.39	.15
	STAIS6	.56	.32
	STAIS7	.57	.32
	STAIS8	.56	.32
	STAIS9	.67	.45
	STAIS10	.73	.54
	STAIS11	.62	.38
	STAIS12	.66	.44
	STAIS13	.62	.38
	STAIS14	.46	.21
	STAIS15	.73	.53
	STAIS16	.65	.43
	STAIS17	.57	.32
	STAIS18	.42	.18
	STAIS19	.41	.17
	STAIS20	.72	.52
Generalised Anxiety	GAD1	.76	.57
	GAD2	.77	.59
	GAD3	.73	.54
	GAD4	.68	.46
	GAD5	.56	.31
	GAD6	.50	.25
	GAD7	.50	.25
Maths	AMAS1	.52	.28
	AMAS2	.70	.50

	AMAS3	.67	.44
	AMAS4	.67	.45
	AMAS5	.71	.51
	AMAS6	.65	.43
	AMAS7	.56	.32
	AMAS8	.66	.44
	AMAS9	.66	.43
Social	ASC1	.49	.24
	ASC2	.72	.52
	ASC3	.74	.55
	ASC4	.45	.20
	ASC5	.61	.37
	ASC6	.68	.46
	ASC7	.52	.27
	ASC8	.63	.39
	ASC9	.68	.46
	ASC10	.67	.45
	ASC11	.47	.22
	ASC12	.71	.50
	ASC13	.55	.31
	ASC14	.67	.45
	ASC15	.77	.59
	ASC16	.64	.41
	ASC17	.67	.45
	ASC18	.65	.43
	ASC19	.70	.49
	ASC20	.55	.30
Spatial	SA1	.72	.52
	SA2	.57	.32

SA3	.69	.48
SA4	.49	.24
SA5	.76	.58
SA6	.69	.48
SA7	.63	.39
SA8	.51	.26
SA9	.51	.26
SA10	.60	.36

Notes: Trait - items of State Trait Anxiety Inventory Trait subscale; State - items of State Trait Anxiety Inventory State Subscale; Generalised Anxiety - items of Generalised Anxiety Disorder Questionnaire; Maths - items of Abbreviated Maths Anxiety Scale; Social - items of Appraisal of Social concerns; Spatial- items of Social Anxiety Questionnaire; for factor loadings, values < 0.3 are highlighted in grey; for communality, values < 0.4 are highlighted in grey;
